# Endothelial cell cycle inhibition enables blood vessel maturation to normalize the tumor vasculature

**DOI:** 10.1172/jci.insight.203732

**Published:** 2026-09-10

**Authors:** Shelby R. Cain, Gael Genet, Nafiisha Genet, Jordon W. Aragon, Madeline G. Jackson, Victoria M. Milosek, Mark R. Schwartz, Umadevi Paila, Aleksandra Cwiek, Zaneta Markowska, Nicholas W. Chavkin, Richard J. Price, Andrew C. Dudley, Karen K. Hirschi

**Affiliations:** 1 Department of Cell Biology and; 2 Developmental Genomics Center,; 3 Robert M. Berne Cardiovascular Research Center,; 4 Comprehensive Cancer Center,; 5 Departments of Molecular Physiology and Biophysics, and; 6 Biomedical Engineering, University of Virginia School of Medicine, Charlottesville, Virginia, USA.; 7 Department of Pediatrics, Division of Cardiology, University of Washington School of Medicine, Seattle, Washington, USA.; 8 Center for Developmental Biology and Regenerative Medicine, Seattle Children’s Research Institute, Seattle, Washington, USA.; 9 Radiology and Medical Imaging, and; 10 Microbiology, Immunology and Cancer Biology, University of Virginia School of Medicine, Charlottesville, Virginia, USA.

**Keywords:** Oncology, Vascular biology, Angiogenesis

## Abstract

Dysfunctional tumor vessels promote disease progression, whereas improved function enhances therapeutic delivery. However, current approaches to normalize tumor vasculature have limited efficacy. In vascular malformations, vessels are similarly dysfunctional, with endothelial cell (EC) hyperproliferation impairing arterial-venous specification. These defects are corrected with palbociclib, a cyclin-dependent kinase 4/6 inhibitor (CDK4/6i) that has beneficial effects on tumor and immune cells, but the effects on tumor vasculature are not well characterized. In our studies, murine mammary tumor ECs (TECs) exhibited disrupted cell cycle and specification, and CDK4/6i promoted TEC cycle control, enabling improved tumor vascular function. To investigate transcriptomic changes, we performed single-cell RNA sequencing (scRNA-seq) of treated and untreated tumors, and healthy tissues. CDK4/6i-mediated TEC cycle arrest promoted arterial-venous specification, cellular junctions, and pericyte association, and suppressed glycolytic and immunosuppressive gene expression. These effects were associated with increased vessel perfusion, decreased tumor hypoxia, and a more favorable immune landscape with immunotherapy. In scRNA-seq datasets from patients treated long-term with CDK4/6i, TECs exhibited similar transcriptomic changes associated with arterial-venous specification, pericyte recruitment, and immune signaling. Thus, in contrast to current strategies, CDK4/6i-mediated vascular changes may be maintained with continued treatment, highlighting the relevance of modulating TEC cycle to improve vessel maturation/function.

## Abstract 2

Abnormal tumor blood vessels promote disease. We found that pharmacologically inhibiting endothelial cell cycle enhances tumor vessel maturation/function, reduces hypoxia, and cooperates with immunotherapy.

## Graphical abstract

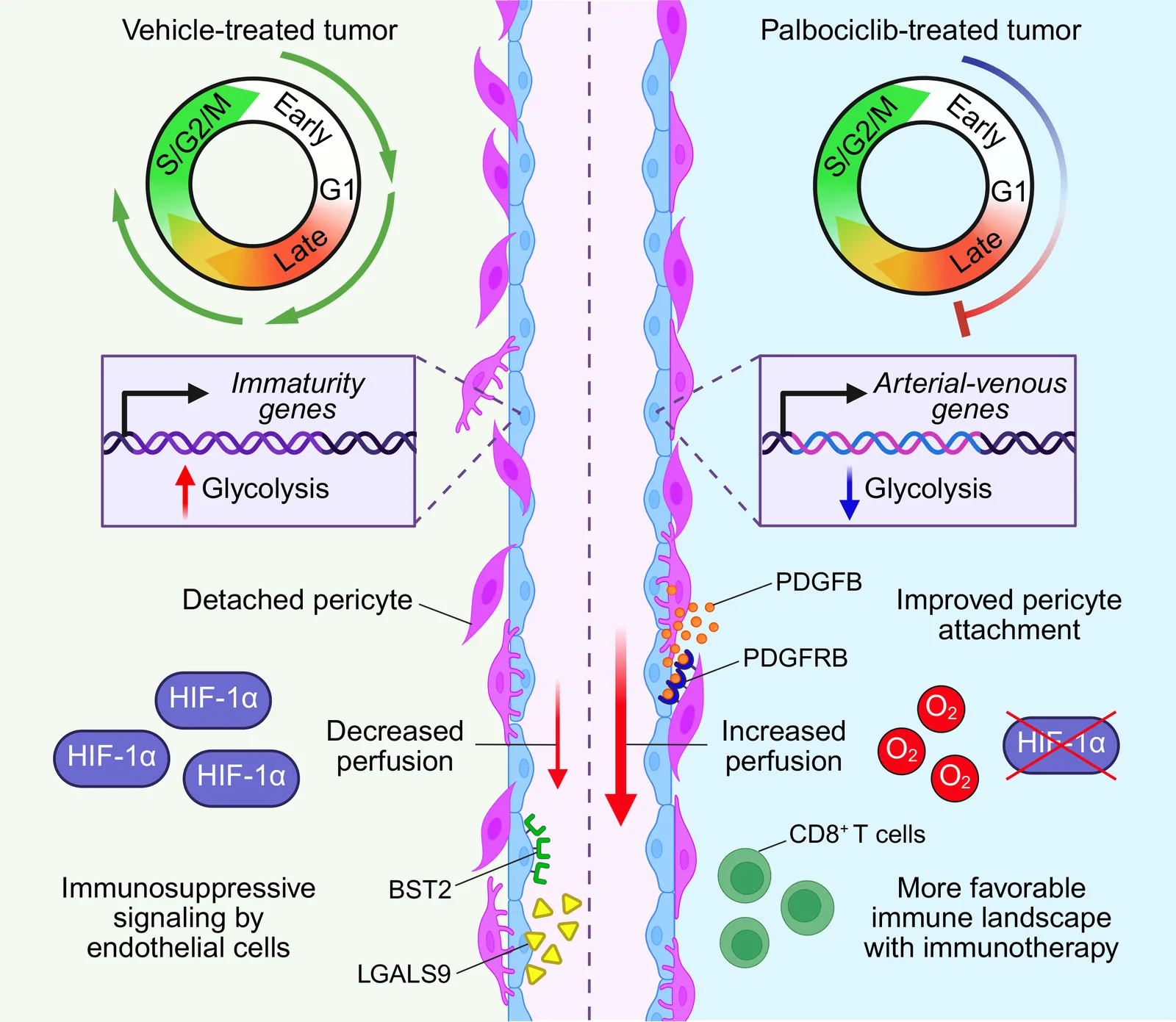

## Introduction

Angiogenesis occurs throughout life but can be pathological in diseases, including cancer (1). In the tumor microenvironment, an imbalance between pro- and antiangiogenic factors results in dysfunctional, unevenly distributed vasculature lacking normal arterial-capillary-venous hierarchies (2–4). Tumor-associated vessels display loose pericyte associations and impaired barrier function, resulting in leaky vessels and high interstitial fluid pressure (2, 5–7). Poor perfusion creates areas of severe hypoxia that select for cancer cells with increased metastatic potential and promote immune suppression via regulatory T (Treg) cell recruitment (8–10). Subsequently, vascular dysfunction impairs the delivery and efficacy of therapeutics (11). Clinical interventions targeting angiogenic factors suggest that normalizing tumor vascular structure and function increases drug delivery and efficacy (12–14). However, antiangiogenics typically result in a small window of efficacy due to resistance or over-pruning (15–17). Thus, there is a clinical need for methods to normalize vasculature without these limitations for more effective cancer treatment.

Arterial-venous specification, as well as pericyte coverage of vessels, are key steps in vessel maturation (18, 19). In our vascular development studies, using the fluorescent ubiquitination-based cell cycle indicator Fucci2 (20–22), we found that endothelial cell (EC) cycle control is required for arterial-venous specification (23, 24). That is, ECs residing in early G_1_ respond to signals promoting venous specification, while ECs in late G_1_ respond to signals promoting arterial specification (23, 24). In mouse models of hereditary hemorrhagic telangiectasia, a disease characterized by disordered and leaky vessels that resemble tumor vessels, we found dysregulated EC cycle disrupts arterial-venous specification and vessel maturity. Importantly, treatment with a cell cycle inhibitor that arrests ECs in G_1_ state prevents these defects (25).

Palbociclib is a cyclin-dependent kinase 4/6 inhibitor (CDK4/6i) that blocks the activity of the CDK4/6–cyclin D1 (CCND1) complex and thereby prevents hyperphosphorylation of retinoblastoma (RB) protein, maintaining repression of genes that promote G_1_ to S phase transition (26–28). Palbociclib, in combination with hormone antagonists, is FDA approved to treat patients with hormone receptor–positive (HR^+^) HER2^–^ advanced or metastatic breast cancer, which relies on estrogen signaling and RB inhibition for proliferation (29–33). Palbociclib is clinically used to target tumor cell cycle progression, but its effects on tumor ECs (TECs) and vascular structure and function are not well understood (34). Herein, we hypothesized that TEC cycle state is dysregulated, leading to impaired specification and vessel maturation and palbociclib-induced TEC cycle arrest will effectively promote vessel maturation and functionality.

In our studies, EO771 mouse mammary TECs exhibited increased proliferation and impaired arterial-venous specification. Palbociclib treatment decreased TEC proliferation and promoted G_1_ arrest, enabling arterial-venous specification, identified by single-cell RNA sequencing (scRNA-seq). Palbociclib-treated TECs exhibited differential expression of genes regulating metabolism, pericyte recruitment, cellular junctions, and immunosuppressive signaling. These effects were associated with increased tumor vessel perfusion, reduced tumor hypoxia, and increased CD8^+^ T cell representation when combined with an anti–PD-1 antibody. In a separate tumor model, D4M melanoma, palbociclib similarly promoted a late G_1_ state and enhanced arterial-venous gene expression in TECs. Importantly, scRNA-seq analysis of TECs from patients with breast cancer after long-term palbociclib treatment showed correlative transcriptomic changes associated with enhanced arterial-venous specification, maturation, pericyte recruitment, and immune signaling. Our collective findings suggest that modulating TEC cycle may promote and sustain tumor vessel maturation across multiple tumor types, with potential clinical applications.

## Results

### TECs are immature, with dysregulated cell cycle states.

To investigate tumor vascular structure and TEC cycle state and specification, we induced mammary tumors by injecting EO771 mammary cancer cells (35) into mammary fat pads and vehicle (HBSS) into contralateral control fat pads of syngeneic C57BL/6J mice (Figure 1A). After 15 days, we perfused mice with Vascupaint for ex vivo imaging, harvested healthy mammary fat pads and tumors, and found EO771 tumors exhibited irregular vessels compared with control tissues (Figure 1B). To further interrogate vascular structure and identity, we immunostained tumors and fat pads for ECs (CD31^+^), and quantified vessel diameter and expression of a venous-enriched protein, endomucin (EMCN) (Figure 1C). Tumor vessels exhibited significantly increased diameter and significantly decreased EMCN expression compared with healthy mammary fat pads, consistent with tumor vessel immaturity and impaired specification (Figure 1, D and E). To determine whether TECs are transcriptionally specified, we separately dissociated mammary fat pads and tumors into single-cell suspensions and isolated ECs (CD31^+^CD45^–^) by FACS from each group for gene expression analysis by quantitative PCR (qPCR). TECs had reduced expression of arterial- (*Sox17*, *Gja4*, *Efnb2*, *Mecom*) (22, 36, 37), venous- (*Nrp2*, *Ephb4*, *Nr2f2*, *Emcn*) (38), and capillary-enriched (*Rgcc*, *Aqp7*, *Car4*) (39, 40) genes, and increased expression of genes associated with proliferation (*Mki67*, *Top2a*), immaturity (*Vwa1*, *Aplnr*) (41, 42), and sprouting (*Apln*) compared with normal mammary ECs (NECs) (Figure 1, F–I), consistent with dysregulated arterial-venous specification and impaired tumor vessel maturation.

Previously, we found a dysregulated EC cycle state impairs arterial-venous maturation during development and disease (23–25). Therefore, we investigated whether dysregulated EC cycle was associated with tumor vessel immaturity. We cryosectioned tumors and mammary fat pads from mice expressing the Fucci2 cell cycle reporter and immunostained for ERG (EC nuclei) (43) to quantify EC cycle states (Figure 2, A and B). While we observed no differences in early G_1_ state, TEC proportions in late G_1_ were significantly decreased and S/G_2_/M significantly increased, indicating dysregulated cell cycle control (Figure 2C) may underly immature tumor vessels.

### CDK4/6i decreases EC proliferation and promotes a late G_1_ state.

We first confirmed that the CDK4/6i palbociclib reduced EC proliferation in vitro using EdU incorporation assays in human umbilical vein ECs (HUVECs) treated with vehicle (DMSO) or CDK4/6i (Supplemental Figure 1A; supplemental material available online with this article; https://doi.org/10.1172/jci.insight.203732DS1). We measured proliferating (EdU^+^) cells using flow cytometry and found CDK4/6i suppressed HUVEC proliferation (Supplemental Figure 1, B and C). Next, we tested how CDK4/6i affected EC cycle states using Fucci-expressing HUVECs (Fucci-HUVECs) (20). By flow cytometric analysis (Supplemental Figure 1D), CDK4/6i increased the proportion of Fucci-HUVECs in late G_1_ and decreased cells in early G_1_ and S/G_2_/M (Supplemental Figure 1E), as previously described (25).

To examine how CDK4/6i affects TEC proliferation in vivo, we treated mice bearing EO771 tumors with palbociclib (CDK4/6i) or vehicle on day 12 (D12), D13, and D14 and administered EdU 12 hours before mammary and tumor harvest (D15) (Supplemental Figure 2A). We isolated cells from these tissues, enriched for CD31^+^ cells with magnetic beads, and analyzed EC (CD31^+^CD45^–^) proliferation (EdU^+^) by flow cytometry (Supplemental Figure 2B). TECs were significantly more proliferative than NECs, and CDK4/6i significantly reduced TEC proliferation (Supplemental Figure 2C).

To determine whether CDK4/6i changes TEC cycle states in vivo, we generated Fucci2 mice bearing EO771 tumors and treated the mice with CDK4/6i following the timeline in Figure 2D. Immunofluorescence quantification of ERG and Fucci2 expression in tumors (Figure 2E) revealed significantly increased proportions of TECs residing in late G_1_ and decreased proportions in S/G_2_/M following CDK4/6i treatment (Figure 2F). Our data suggest CDK4/6i effectively promotes a late G_1_ state, which we found enables arterial specification (23, 24).

We then tested whether CDK4/6i affected proliferation of other cells within the tumor microenvironment. EdU incorporation assays showed that CDK4/6i suppressed EO771 cell proliferation in vitro (Supplemental Figure 3, A and B) and significantly decreased proliferation of non-ECs (CD31^–^) in vivo, although with a smaller fold-change than in TECs (Supplemental Figure 3, C and D). We measured tumor size during treatment and found CDK4/6i significantly decreased tumor size on D15, despite the short treatment window (Supplemental Figure 3E). Overall, TEC proliferation was more sensitive to CDK4/6i versus other tumor cell types.

We assessed whether changes in TEC proliferation persist following cessation of CDK4/6i treatment, by measuring EdU incorporation 1-week after treatment (Supplemental Figure 4A). We found there was no longer a difference in TEC proliferation between vehicle- and CDK4/6i-treated groups (Supplemental Figure 4, B and C), suggesting the effects of CDK4/6i are transient.

To determine whether CDK4/6i changes TEC cycle states in other tumor models, we performed similar studies in Fucci2 mice bearing D4M melanoma tumors (Supplemental Figure 5A). We quantified EC cycle states in tumor sections (Supplemental Figure 5B) on D15 of tumor growth following CDK4/6i treatment on D12, D13, and D14. CDK4/6i-treated D4M TECs displayed significantly increased proportions in late G_1_ and decreased proportions in S/G_2_/M versus controls (Supplemental Figure 5C). These results suggest that cell cycle inhibitors may mature vasculature across tumor types.

### CDK4/6i treatment decreases tumor hypoxia and increases vessel perfusion.

CDK4/6i increased TEC proportions in late G_1_ and decreased proportions in S/G_2_/M (Figure 2), which improves vessel function in disease models (25). Therefore, we investigated whether EC cycle inhibition was associated with functional changes in poorly perfused tumor vessels that exhibit hypoxic areas promoting oncogenic signaling and metastasis (9, 10, 44). To measure tumor hypoxia, we transduced EO771 cells with a hypoxia response element fluorescent reporter (HRE-dUnaG) before inoculation in vivo, and quantified hypoxia-inducible factor (HIF) activity in tumors with and without CDK4/6i (Figure 3A and Supplemental Figure 6A) (45). Fixed tumors were sectioned by vibratome for fluorescence imaging (Figure 3B), and dUnaG intensity (Figure 3C) and dUnaG^+^ percentage tumor area (Figure 3D) were significantly decreased by CDK4/6i, indicating decreased hypoxia. D4M melanoma cells were similarly transduced with HRE-dUnaG, and the resulting tumors trended toward decreased percentage hypoxic area following CDK4/6i (Supplemental Figure 6, B–D).

Since CDK4/6i decreases EO771 tumor size, we investigated whether the decrease in tumor hypoxia is a result of CDK4/6i-mediated tumor volume reduction rather than vascular changes. We performed linear regression analysis of EO771 tumor size versus percentage hypoxic tumor area and found CDK4/6i significantly reduced hypoxia compared with vehicle at equivalent tumor volumes (Supplemental Figure 7, A and B). Thus, CDK4/6i treatment reduced tumor hypoxia independently of reduced tumor volume. To examine whether reduced tumor hypoxia is maintained following CDK4/6i, we treated mice bearing EO771 HRE-dUnaG tumors with and without CDK4/6i for 8 days (Supplemental Figure 7C) and performed fluorescence imaging. The percentage hypoxic tumor area was significantly decreased with extended treatment (Supplemental Figure 7, D and E), indicating CDK4/6i benefits can be maintained.

To determine whether reduced hypoxia was associated with changes in vascular function, we perfused mice bearing EO771 tumors with fluorescent lectin by tail vein injection before euthanasia on D15 to label vessels exposed to blood flow (Figure 3E and Supplemental Figure 8A). We imaged tumor sections for blood vessels (CD31^+^) and lectin fluorescence (Figure 3F) and quantified vessel perfusion as the percentage of vessel area that was lectin-positive, and found perfusion significantly increased with CDK4/6i (Figure 3G). To investigate whether observed changes in vessel perfusion were a consequence of decreased EO771 tumor size after CDK4/6i, we performed linear regression analysis of tumor size versus perfusion for both vehicle- and CDK4/6i-treated groups (Supplemental Figure 8B). Regression line slopes did not differ between groups, indicating CDK4/6i did not change the relationship between tumor size and perfusion. The CDK4/6i-treated group displayed a trend toward increased perfusion across tumor sizes compared with the vehicle-treated group (*P* = 0.068), but the overall weak correlation between EO771 tumor size and perfusion suggests that perfusion variability cannot be attributed only to changes in tumor size. Collectively, our results indicate that CDK4/6i-treated tumors have increased vessel perfusion and decreased hypoxic signaling independent of tumor size, and decreased hypoxia can be maintained with extended treatment.

### CDK4/6i treatment induces TEC expression of late G_1_–enriched genes and reduces expression of proliferation genes.

To assess transcriptomic changes associated with CDK4/6i-mediated improved vessel function in EO771 tumors, we performed scRNA-seq on vehicle-treated NECs and TECs, and CDK4/6i-treated TECs (Figure 4A). We mechanically and enzymatically dissociated vehicle-treated normal mammary tissues, as well as vehicle- and CDK4/6i-treated tumors into single-cell suspensions, enriched each pooled group for CD31^+^ cells with magnetic beads, and FACS-isolated live ECs (PI^–^CD31^+^CD45^–^) (Figure 4B). In TEC samples, cells from the tumor microenvironment were spiked back in to make up approximately 10% of total cells sequenced to enable analysis of EC interactions with other cell types. Seurat was used for quality control and sample integration before dimensionality reduction with UMAP (46–48) (Supplemental Figure 9A). Cell cluster annotations (Figure 4C) were based on known cell markers and labeled as Tumor (*Rhox5*) (49), Immune (*Ptprc*) (50), Pericyte (*Des*) (51), Endothelial (*Cdh5*) (52), and Lymphatic (*Lyve1*) (53) (Supplemental Figure 9B).

Next, we subset TECs from the Seurat object, performed differential gene expression analysis on CDK4/5i-treated TECs, and identified changes in gene expression associated with proliferation, immunosuppression, glycolysis, hypoxia, and leukocyte trafficking (Figure 4D). *Ankrd37*, a target of HIF signaling (54), exhibited significantly lower expression after CDK4/6i treatment, consistent with observed decreased tumor hypoxia. CDK4/6i-treated TECs also had significantly lower expression of proliferation genes *Top2a* and *Mki67*. We further analyzed the relative average expression of cell cycle genes in each group and found trends of increased relative expression in TECs versus NECs, with lower relative expression in TECs after CDK4/6i treatment (Figure 4E), supporting the EdU analysis.

Fucci2 quantification indicated that CDK4/6i increased TECs in late G_1_ and decreased proportions in early G_1_ and S/G_2_/M, and we next determined whether CDK4/6i treatment induced transcriptional signatures corresponding to the observed cell cycle changes. To analyze EC cycle state changes in the scRNA-seq dataset, we identified cell cycle state–specific gene signatures by conducting bulk RNA-seq on FACS-isolated murine Fucci2-expressing NECs in early and late G_1_ (Supplemental Figure 9C). Differential gene expression analysis comparing early versus late G_1_ NECs revealed the top 90 significantly enriched genes in each state (Supplemental Table 1) that were used to generate cell cycle module scores applied to all TECs in our scRNA-seq dataset. Late G_1_ cell cycle module scores did not differ in TECs compared with NECs, but CDK4/6i-treated TECs had significantly higher late G_1_ scores (Supplemental Figure 9D). Early G_1_ cell cycle module scores were significantly higher in TECs versus NECs and significantly reduced in CDK4/6i-treated TECs (Supplemental Figure 9E). Overall, CDK4/6i decreased early G_1_–enriched gene expression and increased late G_1_–enriched gene expression.

### CDK4/6i treatment enhances TEC expression of arterial- and venous-enriched genes.

To assess the effects of CDK4/6i on EC specification, we subset all ECs from the Seurat object and re-clustered them to identify EC subtypes. Analysis of subtype-enriched genes revealed the expected lymphatic, tip, mitotic, venous, cap-venous, capillary, cap-arterial, and arterial EC subtypes (Figure 4F), visualized with UMAP (47, 48) (Supplemental Figure 9F). We then quantified TEC subtype proportions in NECs, TECs, and CDK4/6i-treated TECs (Supplemental Figure 9G). Consistent with other analyses, TECs had lower arterial and venous and higher tip and mitotic proportions than NECs, indicating decreased maturity. We did not observe changes in CDK4/6i-treated TEC subtype proportions.

We next determined whether CDK4/6i treatment enhances specification within EC subtypes. Relative arterial gene expression was lower in arterial TECs versus arterial NECs and higher with CDK4/6i treatment (Figure 4G), and venous genes followed the same trends (Figure 4H). Our results support the idea that TEC cycle inhibition promotes late G_1_ gene expression and enhances subtype-specific gene expression in arterial and venous ECs.

To analyze transcriptomic changes associated with TEC specification in the D4M melanoma model, we FACS-isolated TECs (Supplemental Figure 10A) for qPCR. CDK4/6i-treated D4M TECs had increased expression of arterial- (*Efnb2*, *Sox17*) and venous-enriched (*Ephb4*, *Vwf*) genes and decreased proliferation marker *Mki67* (Supplemental Figure 10, B–D). These results are consistent with our scRNA-seq analysis of EO771 TECs and further support that CDK4/6i increases TEC specification gene expression.

### CDK4/6i treatment decreases EC glycolytic activity.

Glycolytic gene expression (*Pgk1*, *Pkm*, *Eno1*) was significantly lower in TECs following cell cycle inhibition (Figure 4D). Glycolytic regulation is closely linked to EC behavior, as glycolytic activation is important for vessel sprouting (55, 56). We previously found glycolytic gene expression enriched in murine vascular malformations, and CDK4/6i treatment reprogramed this pathological metabolism (25). However, the effects of CDK4/6i on TEC metabolism are not well understood.

Using our scRNA-seq data, we compared average glycolytic gene expression in NECs, TECs, and CDK4/6i-treated TECs. Relative glycolytic gene expression was higher in TECs compared with NECs and decreased following CDK4/6i treatment (Figure 5A). To determine whether this was a direct effect of CDK4/6i on ECs, we performed a glycolytic stress test to measure the extracellular acidification rate (ECAR) of HUVECs that were treated with vehicle or CDK4/6i for 24 hours (Figure 5B). CDK4/6i-treated HUVECs exhibited significantly reduced glycolysis, glycolytic capacity, and glycolytic reserve (Figure 5, C–E), indicating CDK4/6i directly reduces EC glycolytic activity and may normalize pathological TEC metabolism.

We also tested whether CDK4/6i affects additional aspects of cellular metabolism, including mitochondrial respiration, by measuring the oxygen consumption rate (OCR) of vehicle- or CDK4/6i-treated HUVECs (Supplemental Figure 11A). CDK4/6i-treated HUVECs exhibited significantly decreased basal and maximal respiration and ATP production, while non-mitochondrial respiration and spare respiratory capacity were unchanged (Supplemental Figure 11, B–F). Although CDK4/6i reduced mitochondrial respiration, proton leak was significantly decreased and coupling efficiency significantly increased (Supplemental Figure 11, G and H), indicating that mitochondrial respiration is more efficient. Reduced glycolytic and mitochondrial activity indicate decreased metabolic demands (Supplemental Figure 11I), consistent with decreased proliferation.

### CDK4/6i treatment increases expression of genes regulating TEC junctions and pericyte recruitment.

Decreased TEC glycolytic activity is linked to improved vessel barrier function and pericyte coverage (55). Using our scRNA-seq datasets containing TECs and other tumor cell types, CellChat (57, 58) analysis identified ligand-receptor pairs with significantly increased communication probability between TECs and TECs and TECs and pericytes after CDK4/6i treatment. Junctional adhesion molecule 2 (*Jam2*) signaling between TECs was predicted to significantly increase following CDK4/6i treatment (Figure 6A), and *Jam2* is involved in regulating barrier function and leukocyte extravasation at EC junctions (59). Similarly, *Plvap* and *Cd300lg* (Figure 4D), genes regulating leukocyte extravasation, were higher in CDK4/6i-treated TECs. CDK4/6i was also predicted to increase TEC *Pdgfb* to pericyte *Pdgfrb* signaling, a pathway crucial for pericyte recruitment (Figure 6A) (60). We examined genes regulating TEC junctions and vessel stability and found trends of decreased relative gene expression in TECs compared with NECs, and higher expression in CDK4/6i-treated TECs, including *Cdh5* and *Pecam1* (Figure 6B). Thus, scRNA-seq analysis suggests cell cycle inhibition enabled expression of genes regulating vessel barrier formation and/or function. We also examined D4M melanoma TEC gene expression and found significantly increased *Jam2*, and modestly increased *Cdh5* and *Plvap* (Supplemental Figure 12, A and B), consistent with transcriptomic changes regulating barrier and leukocyte trafficking in CDK4/6i-treated EO771 TECs.

Tight pericyte-vessel associations are important for vessel stability, maturity, and barrier function (5, 18, 51); consequently, the loose pericyte-vessel associations in tumors contribute to vessel dysfunction (2, 6). Therefore, we investigated whether increased TEC *Pdgfb* expression is associated with changes in pericyte-vessel associations. We immunostained pericytes (NG2^+^) and ECs (CD31^+^) in vehicle- and CDK4/6i-treated EO771 tumors (Figure 6, C and D), measured the percentage of vessel area colocalized with pericytes and NG2 intensity on vessels, and found no CDK4/6i-induced changes (Supplemental Figure 13, A–C). In further analysis, we examined tumor pericyte-vessel associations by measuring angles of pericyte detachment from vessels, with smaller angles indicating tighter pericyte-vessel associations (Figure 6E). We averaged the angle of detachment per tumor to calculate an orientation index that significantly increased with CDK4/6i, indicating that although overall coverage was unchanged, pericytes were more tightly associated with vessels (Figure 6F). We also measured the relative NG2^+^ area detached from vessels compared with NG2^+^ area attached to vessels (Figure 6G), and relative detachment was significantly decreased after CDK4/6i (Figure 6H). Our collective results suggest CDK4/6i treatment augments gene expression associated with pericyte recruitment and TEC junctions and reduces pericyte detachment from tumor vessels.

Pericyte associations improved after 3 days of treatment, and we hypothesized that longer treatment could result in improved pericyte coverage. After extended (8 days) CDK4/6i treatment, the percentage of vessel area colocalized with NG2 was significantly increased (Supplemental Figure 13, D–F). Thus, CDK4/6i improves pericyte coverage of vessels following reduced pericyte detachment. We also examined pericyte coverage in D4M tumors with and without CDK4/6i (for 3 days) and found a trending increase in the percentage of vessel area colocalized with NG2 (Supplemental Figure 13, G and H), indicating CDK4/6i may improve pericyte coverage at different rates in the 2 models.

### CDK4/6i decreases immunosuppressive signaling mediated by TECs.

Using scRNA-seq analysis, we found that *Bst2*, an oncogene associated with an immunosuppressive tumor microenvironment (61), significantly decreased in CDK4/6i-treated EO771 TECs (Figure 4D). Endothelial BST2 mediates monocyte adhesion, and BST2 signaling induces M2 polarization in macrophages (62, 63). We validated changes in TEC BST*2* protein expression by quantifying TEC membrane (CD31^+^) colocalization with BST2 in vehicle- or CDK4/6i-treated EO771 tumors (Figure 7, A and B). CDK4/6i significantly decreased the percentage of area of TEC membrane expressing BST2 (Figure 7C). We then analyzed macrophage polarization–associated gene expression in our scRNA-seq data (Supplemental Figure 14, A and B). In CDK4/6i-treated tumor macrophages, proinflammatory M1-associated gene expression (*Cd86*, *Cxcl9*, *Il1b*) (64) was significantly increased (Supplemental Figure 14C), suggesting an environment with reduced immunosuppressive signaling. Thus, decreased TEC BST2 may be a mechanism by which CDK4/6i decreases tumor immunosuppression. M2-associated genes were unchanged (*Mrc1*, *Tgfb1*) or increased (*Chil*3) (Supplemental Figure 12D); thus, CDK4/6i may have complex effects on tumor-associated macrophages.

Although CDK4/6i was shown to impact tumor immunity via effects on immune and tumor cells (65–67), its effects on TEC immune signaling are not well understood. We performed CellChat (57, 58) analysis to identify signaling changes between TECs and immune cells. Interactions predicted to decrease with CDK4/6i were immunosuppressive signaling pathways, including TEC *Lgals9* signaling to immune cell *Cd44* and *Havrc2* (Figure 7D). *Cd44* promotes Treg cell phenotypes (68) and *Havrc2* encodes immune checkpoint inhibitor TIM-3 that suppresses effector T cells (69, 70). Thus, key immunosuppressive signaling pathways were predicted to be dampened in TECs following cell cycle inhibition, supporting decreased immune tolerance in the tumor microenvironment.

### CDK4/6i cooperates with anti–PD-1 immunotherapy.

Given that CDK4/6i treatment decreased hypoxic signaling and increased vessel perfusion in EO771 tumors and was predicted to alter TEC–immune cell communication, we hypothesized that CDK4/6i-modulated vessels could cooperate with immunotherapy. To test this, we incorporated anti–PD-1 antibody treatment into our tumor model (Figure 7E) and treated groups with vehicle, CDK4/6i, anti–PD-1, or combination treatments. During this treatment timeline, tumor growth was not significantly different between groups (Supplemental Figure 15, A and B). We then analyzed changes in the immune landscape to evaluate potential cooperation of combined treatments and focused on cytotoxic CD8^+^ T cells that mediate antitumor immune responses (71, 72). We quantified CD8 density in tumor sections (Figure 7F and Supplemental Figure 15C) at different distances from the tumor edge and found the tumor core had significantly more CD8^+^ cells in the combination treatment group versus controls (Figure 7G), indicating combined treatment enhances CD8^+^ T cell representation in the tumor microenvironment.

To more rigorously quantify changes in antitumor immune responses, we used flow cytometry (Supplemental Figures 16 and 17). We measured the ratio of cytotoxic (CD8^+^) T cells to Tregs (CD4^+^FOXP3^+^) (73, 74). Combination therapy significantly increased the CD8^+^/Treg cell ratio compared with control and anti–PD-1–treated groups (Figure 7H), whereas neither anti–PD-1 nor CDK4/6i monotherapy significantly affected this ratio compared to controls. Thus, combined CDK4/6i and anti–PD-1 therapies improved the T cell immune landscape in EO771 tumors.

### scRNA-seq of human metastasized breast cancer from CDK4/6i-treated patients reveals similar transcriptomic changes in TEC signaling and specification.

To investigate the clinical relevance of our results, we analyzed publicly available human scRNA-seq data from Luo et al. (75). The dataset consists of CDK4/6i-treated patients with HR^+^HER2^–^ metastatic breast cancer who had biopsies of liver metastasis analyzed by scRNA-seq. We analyzed samples from 5 patients biopsied before CDK4/6i treatment (baseline), and 2 patients biopsied after palbociclib combined with letrozole or ZN-c5 (hormone therapies). scRNA-seq data from patient ECs treated with CDK4/6i as a monotherapy were not available. Therefore, we analyzed CDK4/6i+letrozol– and CDK4/6i+ZN-c5–treated patient data separately to account for hormone therapy–specific effects.

We visualized the integrated human breast cancer scRNA-seq dataset and subset ECs based on *CDH5* expression (Figure 8, A and B). Although TEC numbers were too low to resolve subtypes, we analyzed relative expression of arterial- and venous-enriched genes for all TECs in baseline, and CDK4/6i+letrozole or CDK4/6i+ZN-c5 groups (Figure 8, C and D). These genes were then used to calculate arterial and venous module scores per TEC. CDK4/6i+letrozole TECs had significantly increased arterial scores compared with baseline TECs, and CDK4/6i+ZN-c5 TECs trended towards increased arterial scores (Figure 8E). Additionally, both CDK4/6i-treated groups had significantly increased venous module scores compared with baseline TECs (Figure 8F). Overall, the trends in human breast cancer EC data paralleled our findings that CDK4/6i treatment enhances TEC arterial-venous specification in mammary tumors (Figure 4, G and H).

We next analyzed changes in human TEC signaling to immune cells. In mice, CDK4/6i treatment significantly decreased both TEC *Bst2* gene expression and the percentage of vessel area that was BST2^+^, indicating reduced immunosuppressive signaling (Figure 7, A–C). In human scRNA-seq data, *BST2* expression was significantly decreased in CDK4/6i-treated TECs compared with baseline, suggesting CDK4/6i may decrease BST2 immunosuppressive signaling in humans, consistent with our EO771 model (Figure 8G).

Finally, we investigated changes in human TEC-pericyte communication after CDK4/6i treatment. In our mouse models, CellChat (57, 58) analysis predicted TEC *Pdgfb* to pericyte *Pdgfrb* signaling was significantly more probable after CDK4/6i treatment, and this was associated with increased TEC *Pdgfb* gene expression and improved pericyte-vessel attachments (Figure 6). Correspondingly, in humans, CDK4/6i treatment significantly increased TEC *PDGFB* expression compared with baseline TECs (Figure 8H), suggesting it may also improve TEC-pericyte communication therein. In summary, our analysis of human clinical data correlates with our findings in mouse models, supporting clinical potential for palbociclib to have beneficial effects on tumor vasculature.

## Discussion

In our studies, mouse mammary cancer EO771 TECs exhibited impaired arterial-venous specification and dysregulated cell cycle control. Palbociclib (CDK4/6i) treatment improved both mammary and D4M melanoma TEC cycle control and enabled increased expression of arterial- and venous-enriched genes, as well as genes regulating EC junctions that are associated with improved pericyte-vessel associations. In EO771 tumors, TEC cycle inhibition also decreased glycolytic gene expression and enhanced immune cell signaling, and these collective changes were associated with increased vessel perfusion, reduced tumor hypoxia, and improved immune landscape with anti–PD-1 immunotherapy. CDK4/6i treatment also reduced hypoxia in the D4M melanoma model, indicating functional effects consistent across tumor types. Analysis of human breast cancer scRNA-seq data parallels our results, supporting the potential for clinical translation.

In previous studies of vascular development and disease (23, 25), we found that ECs are permissive to venous signals during early G_1_ and to arterial signals during late G_1_ (23, 24). Correspondingly, we found that CDK4/6i increased the proportion of TECs in late G_1_ and enhanced arterial gene expression in arterial TECs. Surprisingly, although CDK4/6i decreased the proportion of TECs in early G_1_, it enhanced venous gene expression in venous TECs. This finding suggests that, due to decreased transition into S/G_2_/M, TECs remain in the G_1_ state, and this may enable both arterial and venous specification.

Downstream of CDK4/6 activity, E2F transcription factors upregulate genes involved in glycolysis (76). Correspondingly, we found CDK4/6i decreased HUVEC glycolytic activity and reduced glycolytic gene expression by TECs to normalize metabolism. Dysfunctional TECs are highly reliant on glycolysis, and targeting glycolysis increases murine tumor vessel maturity and pericyte coverage, resulting in stabilized vessels and decreased metastasis (55, 56, 77, 78). Consistent with this, CDK4/6i significantly improved pericyte-vessel associations and augmented expression of TEC junctional genes; thus, CDK4/6i-mediated decreased EC glycolytic activity may improve vascular structure and function.

Although palbociclib potently inhibited TEC proliferation, non-EC proliferation was also decreased. Therefore, we cannot attribute all vascular and tumor microenvironment changes solely to CDK4/6i-mediated effects on TECs. To further establish a causal role for TEC cycle inhibition, additional studies using EC-specific genetic models are required. To elucidate other possible mechanisms of CDK4/6i treatment, we analyzed changes in signaling among all tumor cell types. TEC communication with immune cells was predicted to be significantly affected by CDK4/6i treatment, consistent with previous studies showing it promotes antitumor immunity through effects on immune and cancer cells (79). Specifically, CDK4/6i was found to enhance interferon signaling, antigen presentation, and CD8^+^ T cell activation, while selectively inhibiting Treg proliferation (67). Our studies indicate TEC cycle inhibition was associated with increased expression of genes regulating immune cell trafficking and decreased immunosuppressive signaling, consistent with immune-permissive vessels. However, CDK4/6i alone did not significantly increase the CD8^+^/Treg cell ratio, suggesting CDK4/6i-mediated changes may require longer treatment or additional therapy to produce functional changes in the tumor immune landscape. The CD8^+^/Treg cell ratio significantly increased only with combined CDK4/6i and anti–PD-1 treatment, suggesting CDK4/6i-treated TECs may contribute to beneficial changes in the tumor immune landscape. CDK4/6i reduced immunosuppressive signaling by TECs and improved vascular function while alleviating tumor hypoxia, which is immunosuppressive (10, 44). Therefore, we propose that CDK4/6i-mediated improved vascular structure and function enabled anti–PD-1 antibody delivery and enhanced CD8^+^ T cell representation in the tumor microenvironment.

Targeting TEC cycle to enable maturation could result in a larger window of efficacy than antiangiogenic therapeutics. In human scRNA-seq data, patient TECs exhibited increased arterial-venous gene expression compared with baseline after 8–9 months of CDK4/6i treatment, indicating sustained effects on TEC specification. However, conclusions from this dataset are limited by small patient numbers, low TEC numbers, and potentially confounded by hormone therapy. Additionally, biopsy samples were taken at the time of cancer progression and resistance to CDK4/6i treatment. Nonetheless, continued expression of EC specification genes may support vascular function and delivery of therapeutics at this time point.

Abnormalities in the tumor-associated vasculature enable tumor progression and invasion and impair responses to immunotherapies and chemotherapy (11–14, 56). Thus, there is a clinical need for effective methods to normalize the tumor vasculature. Further understanding the role of TEC cycle dysregulation in tumor vasculature, and the effects of its restoration, could lead to strategies to mature tumor blood vessels. In support of this idea, CDK4/6i improved vascular function and may be a tool for enhanced therapeutic delivery. Additionally, our findings were not confined to one mouse tumor model. Consistent with our findings in EO771 mammary tumors, CDK4/6i promoted late G_1_ and expression of arterial- and venous-enriched genes in D4M TECs. Thus, our studies suggest that cell cycle inhibitors could be used to mature the vasculature more broadly across tumors and future studies are essential to test additional tumor models, as well as optimize the timing of palbociclib treatment in combination with other therapeutics.

## Methods

### Sex as a biological variable.

For mammary cancer studies, we cannot eliminate sex as a biological variable because we only used females; this is important to consider with interpretation of results. All melanoma studies included males and females in vehicle and CDK4/6i treatment groups, and CDK4/6i did not significantly affect tumor growth compared to controls in males or females, analyzed independently.

### Cell culture.

HUVECs (Lonza, C2519AS) were cultured in EGM 2-Bulletkit medium (Lonza, CC-3156 and CC-4176) and used at low passages (P1–P6). EO771 (ATCC, CRL-3461), HEK293T/17 (ATCC, CRL-11268), and D4M-3A cells (provided in-house) were cultured in high-glucose DMEM (Gibco, 11965092), 10% heat-inactivated FBS, and 1% antibiotic-antimycotic at 37°C in 5% CO_2_.

### Glycolytic and mitochondrial stress tests.

Glycolytic function was assessed by ECAR using a glycolytic stress test. HUVECs were seeded onto a Seahorse XFe 96-well plate (Agilent Technologies) at 10,000 cells/well in EGM 2 (Lonza, CC-3156 and CC-4176) and treated with DMSO or palbociclib (2 μM) 24 hours before testing. ECAR was first measured in the absence of glucose (DMEM containing 2 mM glutamine; pH = 7.35 ± 0.01 at 37°C). After 3 baseline ECAR measurements, glucose (20 mM), oligomycin (1 μM), and 2-deoxy-D-glucose (2-DG; 80 mM) were sequentially injected to assess glycolytic function, maximal glycolytic capacity, and non-glycolytic acidification, respectively (Sigma-Aldrich). Measurements were collected in 3 cycles of 3 minutes mixing and 3 minutes measure. Glycolytic capacity was calculated by taking the average maximum ECAR after oligomycin injection minus the average last measurement before glucose injection. Respiratory capacity was assessed by OCR using a mitochondrial stress test. First, cells were switched to DMEM with pyruvate (containing 20 mM glucose, 2 mM glutamine; Thermo Fisher Scientific, pH = 7.35 ± 0.01 at 37°C). Following basal OCR measurements (3 cycles of 3 minutes mix and 3 minutes measure), oligomycin (1 μM; Sigma-Aldrich), BAM15 (2 μM; Cayman Chemical), and antimycin A/rotenone (1 μM/100 nM; Sigma-Aldrich) were sequentially injected and 3 measurements taken to assess ATP-linked respiration, maximal respiratory capacity, and non-mitochondrial respiration, respectively. Respiratory capacity was calculated by taking the maximum OCR after BAM15 injection minus the non-mitochondrial respiration. The energy capacity map represents metabolic potential using glycolytic and respiratory capacities.

### EO771 HRE transduction.

NEB bacteria containing pLenti-HRE-dUnaG (45) (Addgene, 124372) were grown overnight in LB broth with 100 μg/mL ampicillin at 30°C. HRE-dUnaG plasmid was isolated with a PureLink HiPure plasmid filter maxiprep kit (Invitrogen, K210017). HEK293T/17 cells were transfected with HRE-dUnaG, psPAX2, and pMD2.G using Lipofectamine 3000 (Invitrogen, L000008). Lentiviral supernatants were harvested after 48 hours and stored at –20°C. Supernatants were mixed 1:1 with cell culture media and centrifuged (800*g*, 5 minutes) to remove debris. EO771 and D4M cells were transduced for 48 hours and selected with puromycin for approximately 2 weeks (EO771, up to 10 μg/mL; D4M, up to 4 μg/mL) then maintained in 1 μg/mL puromycin. HRE-dUnaG EO771 cells were validated using CoCl_2_ treatment (50 μM, 36 hours) and fluorescence imaging.

### HUVEC-Fucci transduction.

Cellomics Technology prepared Fucci lentivirus using the Fast-FUCCI plasmid (pBOB-EF1-FastFUCCI-Puro, Addgene, 86849). HUVECs infected with lentivirus were selected using puromycin (1 μg/mL; Sigma-Aldrich, P9620) and lentiviral transduction was validated using Leica Thunder imaging. pBOB-EF1-FastFUCCI-Puro was a gift from Kevin Brindle and Duncan Jodrell (Cancer Research UK, Cambridge, United Kingdom) (Addgene, 86849; http://n2t.net/addgene:86849; RRID:Addgene 86849).

### In vitro proliferation assays.

Fucci-HUVECs or HUVECs were seeded at 70,000 cells/well on a 6-well plate. After 24 hours, Fucci-HUVECs were treated with palbociclib (1.5 μM) or DMSO and analyzed with flow cytometry (BD Accuri C6) to quantify cell cycle states (early G_1_ = mCherry^–^mVenus^–^, late G_1_ = mCherry^+^mVenus^–^, S/G_2_/M = mCherry^–^mVenus^+^). HUVECs were treated with a range of palbociclib doses and incubated with EdU (5 μM) for 2 hours before cells were harvested, fixed and permeabilized. We used the Click-iT EdU cell proliferation kit (Invitrogen, C10340) to label EdU with Alexa Fluor 647 (AF647) for flow cytometric quantification of proliferation (EdU-AF647+).

### Animal experiments.

R26p-Fucci2 mice (Fucci2, C57BL/6N background) were acquired from Riken (accession, CDB0203T). For WT studies, C57BL/6N mice were purchased from The Jackson Laboratory (strain 000664). For the mammary cancer model, 7- to 9-week-old mice were injected with 5 × 10^5^ EO771 cells in 100 mL HBSS in the fourth mammary fat pad and with vehicle in the contralateral mammary fat pad on D0. For the melanoma model, 7- to 9-week-old mice were subcutaneously injected with 5 × 10^5^ D4M cancer cells in 100 mL HBSS. Mammary tumors and fat pads or melanoma tumors were harvested on D15 of tumor growth.

For CDK4/6i (Selleck, S4482; Sigma-Aldrich, PZ0383) studies, mice received palbociclib (150 mg/kg/day) or vehicle (10% DMSO in PBS) by oral gavage daily from D12–D14 (harvest D15) or D11–D18 (harvest D19 or D21). For anti–PD-1 (BioXCell, BP0146) and CDK4/6i combination studies, mice received palbociclib via oral gavage on D12–D14 and D16, and anti–PD-1 (15 mg/kg in PBS) by intraperitoneal (i.p.) injection on D13, D15, and D17, with harvest on D18. Mice received monotherapy or combination treatment, with vehicle for oral gavage and IgG2a isotype control (15 mg/kg; BioXCell, BP0089) for i.p. injection. Calipers were used to measure tumor volumes, calculated as (length × width^2^)/2.

### Vascular tracer experiments.

Mice received *Lycopersicon* *esculentum* (tomato) lectin, fluorescein (FITC) via tail vein injection (100 μL, 0.5 mg/mL; Thermo Fisher Scientific, L32478) 20 minutes prior to euthanasia. To visualize blood vessels, lethally anesthetized mice received sequential intracardiac injections of PBS with 2 mM EDTA (Ca^2+^ chelator) and 10 U/mL heparin, 4% paraformaldehyde (PFA), and green Vascupaint (ediLumine, SKU MDL-122). Tissues were postfixed with 4% PFA overnight and dehydrated with successive 24-hour methanol incubations (30%, 60%, 100%). Tissues were cleared with benzyl benzoate/benzyl alcohol (1:1) and imaged with bright-field microscopy.

### Genotyping.

Mouse ear clippings were digested in alkaline lysis buffer (25 mM NaOH, 0.2 mM EDTA, pH 12) at 95°C for 30 minutes before the addition of neutralization buffer (40 mM Tris-HCl, pH 5.0). EmeraldAmp GT PCR Master Mix (Takara, RR310B) and mVenus primers from The Jackson Laboratory (Supplemental Table 2) were used for the PCR reaction with annealing temperature 56.1°C. The Fucci2^+^ band is 200 bp and the internal positive control, the interleukin-2 gene, is 300 bp.

### Immunostaining tissues.

For Fucci2, EMCN, and CD8a analysis, cryosectioned tissues were fixed in 4% PFA for 24 hours, cryoprotected in 30% sucrose for 24 hours, embedded in OCT, and stored at –80°C. Serial sections of mammary fat pad (25 μm) and tumor (8 μm) were warmed, rehydrated with PBS, and incubated in methanol (–20°C) for 20 minutes. Tissues were blocked in 5% normal donkey serum or 10% BSA with 1% Triton X-100 for 12 hours. Tissues were incubated overnight at 4°C with primary antibodies (Supplemental Table 3) and with corresponding secondary antibodies for 1 hour (Supplemental Table 4). Tissues were washed 5 times, 3 minutes each, between steps and mounted in fluorescent mounting medium (Agilent Technologies, S3023).

For perfusion, hypoxia, NG2, and BST2 analysis, fixed tumors were embedded in 2% agarose and serially sectioned (200 μm) by vibratome (Leica, VT1000S). Tissues were permeabilized for 1.5 hours in 1% Triton X-100 before blocking in 10% BSA with 1% Triton X-100 for 3 hours. Tissues were immunostained overnight at 4°C with primary antibodies (Supplemental Table 3) and with corresponding secondary antibodies overnight at 4°C (Supplemental Table 4). Hoechst was used to counterstain cell nuclei. Tissues were washed 3 times, 10 minutes each, with 0.1% Triton X-100 in PBS between steps. The Leica DMi8 LIGHTNING confocal microscope and LASX software were used for imaging.

### Image morphometric analysis.

In Fucci2 tissue sections, ECs (ERG^+^) in regions of interest (ROI) were quantified for mCherry^+^ and mVenus^+^ to determine cell cycle states. EO771 and D4M tumors were used as negative controls for HRE-dUnaG image acquisition. Confocal images of HRE-dUnaG–expressing tumors were thresholded to eliminate background in ImageJ (v1.54a, NIH) before tracing the whole tumor section to measure dUnaG^+^ percentage area and intensity. Percentage vessel EMCN^+^ area was quantified using a custom ImageJ macro measuring percentage CD31 colocalized with EMCN.

Tissues from mice not perfused with lectin were used as negative controls for imaging lectin^+^ vessels in vascular tracer experiments. Vessels were traced using ImageJ tools and these ROIs were merged for measurement of lectin perfusion. For perfusion and NG2 measurements, background was subtracted using the rolling ball algorithm (50–60 pixels) in ImageJ before measurements to correct for uneven background fluorescence.

Custom ImageJ macros were used to quantify NG2/CD31 colocalization, NG2 intensity, and relative pericyte detachment. Vessels (CD31^+^) were identified using adaptive thresholding and morphological processing to create vessel ROIs. NG2^+^ signal was thresholded and filtered for contiguous structures (≥0.5 μm^2^) to remove noise. Colocalized NG2^+^ and CD31^+^ signals were measured to calculate pericyte coverage. NG2^+^ signal was measured within 5 μm of vessel ROIs. Contiguous NG2^+^ signal was classified as vessel-attached (0–5 μm from vessel) or vessel-detached (5–15 μm from vessel) when NG2/CD31 colocalization was 5% or greater. Relative pericyte detachment was the ratio of vessel detached to attached NG2^+^ area. Pericyte angle of detachment was manually measured in ImageJ, with only acute angles measured because directionality could not be determined.

The RG2B Colocalization plugin (copyright 2004, Christopher Philip Mauer) in ImageJ replaced overlapping red (BST2) and green (CD31) pixels with blue to indicate colocalization. In ROIs, area of colocalization was divided by vessel area to determine the percentage of BST2^+^ vessel area.

CD8 density and percentage of tumor cells were calculated using custom ImageJ macros that identified CD8^+^ cells as CD8^+^ areas around nuclei (Hoechst). Tumor edge was manually traced and used to calculate distance from edge in the macro.

### qPCR analysis.

Tumors and fat pads were harvested, minced for 3 minutes, and digested enzymatically with Dispase (0.25 U/mL; Worthington Biochemical, LS02104), collagenase type II (0.9 mg/mL; Worthington Biochemical, LS004176), and DNase (7.5 × 10^–3^ mg/mL; Worthington Biochemical, LS002006) in HBSS. Tissues were digested for 1 hour at 37°C, gently pipetted up and down, and incubated another 30 minutes before rinsing with FACS buffer (0.5% BSA, 0.5 M EDTA in PBS). Cells were spun (1000*g,* 10 minutes) and supernatant containing the fat layer was removed. Cells were resuspended in FACS buffer and filtered through Falcon cell strainers (70 μm, Corning, 352350) and spun (300*g*, 5 minutes). Cells were incubated with red blood cell lysis buffer (Thermo Fisher Scientific, BDB555899) for 10 minutes, spun (300*g,* 5 minutes), and resuspended in FACS buffer. Single-cell suspensions were stained for CD31 and CD45 (Supplemental Table 5) and strained into FACS tubes (Genesee, 28-154). Bone marrow was collected for single-stain controls and compensation. ECs (CD31^+^CD45^–^) were isolated by FACS with the BD FACSMelody, lysed in RLTplus (1% β-mercaptoethanol) for RNA extraction using the RNeasy plus micro kit (Qiagen, 74034). Total RNA was measured by Nanodrop to ensure equal cDNA quantities were used for qPCR reactions. RNA was converted into cDNA using a high-capacity cDNA reverse transcription kit (Applied Biosystems, 43-688-13). Target genes were amplified with PowerUp SYBR Green master mix and analyzed with QuantStudio 6 Flex real-time PCR. Gene expression was normalized to β-actin during qPCR analysis.

### EdU incorporation.

Mice were i.p. injected with EdU (200 μL, 5 mg/mL) 12 hours before tumor and fat pad harvest. Tissues were minced (3 minutes) in digestion buffer, mechanically dissociated in C-tubes (Miltenyi Biotec, 130-093-237 and 130-093-235), and enzymatically digested at 37°C (80 rpm, 40 minutes). Cells were rinsed with FACS buffer with 0.5% BSA (Miltenyi Biotec, 130-091-376 and 130-091-222), washed, filtered, and incubated with red blood cell lysis buffer. Cells were incubated (15 minutes, 4°C) in anti-CD31 magnetic beads (Miltenyi Biotec, 130-097-418). Samples were enriched for CD31^+^ cells with magnetic columns (Miltenyi Biotec, 130-042-201) according to the manufacturer’s protocol. EdU was labeled with AF647 using the Click-iT EdU cell proliferation kit (Invitrogen, C10340). Briefly, ECs (CD31^+^CD45^–^) were fixed in 4% PFA (15 minutes), permeabilized in 0.5% Triton X-100 (20 minutes), and incubated in Click-iT reaction cocktail (30 minutes). Cells were stained with fluorescent antibodies against CD31 and CD45 (Supplemental Table 5). Between steps, cells were washed in 1% BSA and spun (300*g*, 5 minutes). Cell proliferation (EdU-AF647^+^) was analyzed via flow cytometry (BD Accuri C6) and FlowJo software (BD Life Sciences; https://www.flowjo.com).

### Sample preparation for bulk RNA-seq.

Fucci2-expressing 9- to 11-week-old mice were euthanized and fourth mammary fat pads from 5 mice were pooled, performed in triplicate. Mammary fat pads were harvested, minced (3 minutes) in digestion buffer, dissociated mechanically, and enzymatically digested at 37°C (80 rpm, 40 minutes). Single-cell suspensions were filtered, washed with FACS buffer, spun (300*g*, 10 minutes), and incubated in red blood cell lysis buffer for 5 minutes. Cells were washed and immunostained with BV421-labeled anti-CD31 and PE-Cy7–labeled anti-CD45 antibodies (Supplemental Table 5). CD31^+^CD45^–^mCherry^–^mVenus^–^ (early G_1_) and CD31^+^CD45^–^mCherry^+^mVenus^–^ (late G_1_) ECs were sorted into buffer RLTplus with RNase inhibitor (114 U/mL) (Roche, 03335399001). DNA was eliminated with RNase-free DNase (Qiagen, 79254) and RNA extracted using the RNeasy plus micro kit (Qiagen, 74034). RNA was eluted in nuclease-free water and frozen at –80°C before shipping to Novogene for low-input bulk mRNA sequencing (RNA amplification with SMARTer kit, sample QC, poly-A enrichment library prep, and NovaSeq PE150).

### Bulk RNA-seq analysis.

The sequence quality of raw fastq files was checked with the FastQC tool (https://github.com/s-andrews/fastqc). TrimGalore (https://github.com/FelixKrueger/TrimGalore) was used for adapter trimming of the Nextera adaptor sequence content that was identified during QC. A STAR index (https://alexdobin.github.io/STAR/) was built for the mouse reference genome (version GRCm39), using gencode gene annotation file (vM36) with a “sjdbOverhang” parameter equal to 150. The adaptor trimmed reads were mapped to the mouse genome reference using the STAR aligner and the mRNA was quantified. The raw counts were used for downstream analysis of the data in R (https://www.r-project.org/). After ensuring proper separation of the early and late G_1_ experiments in PC1 of the PCA plot, differential expression of the 2 datasets was performed with DESeq2 (https://bioconductor.org/packages/DESeq2/). Genes with adjusted *P* values (Benjamini- Hochberg method for controlling FDR) of 0.05 or less were included in downstream analysis.

### Sample preparation for scRNA-seq.

Tumors and fat pads from mice with and without CDK4/6i treatment were harvested, pooled according to tissue and treatment type, and minced (3 minutes) in digestion buffer (described in qPCR analysis) in 2 independent experiments. Tissues were dissociated mechanically and enzymatically digested at 37°C (80 rpm, 40 minutes). Single-cell suspensions were filtered, washed with FACS buffer, and spun (300*g*, 10 minutes) before red blood cell lysis. Cells were incubated with anti-CD31 magnetic beads, BV421-labeled anti-CD31, and PE-Cy7–labeled anti-CD45 antibodies (Supplemental Table 5) for 15 minutes on ice. Samples were enriched for CD31^+^ cells with Miltenyi Biotec MS columns and OctoMACS separator. PI (1:400) was added and live ECs (CD31^+^CD45^–^PI^–^) were sorted into low-glucose DMEM/10% FBS. Live tumor cells from original single-cell suspensions were added to make up approximately 10% of the TEC samples. Cells were washed and resuspended in PBS with 0.04% BSA.

### Single-cell library preparation and scRNA-seq.

The University of Virginia (UVA) Genome Analysis and Technology Core (RRID:SCR_018883) generated single-cell indexed libraries using the 10X Genomics Chromium X platform and the Chromium GEM-X Single Cell 3′ Chip Kit. Two biological replicates per condition with 80%–95% cell viabilities were used. Amplified cDNA was generated following the manufacturer’s protocols for the single-cell GEM-X kit and quality assessed using the Agilent 4200 TapeStation Instrument and the Agilent D5000 HS kit. Single-cell libraries were prepared from the cDNA following the manufacturer’s protocols. Individual libraries were sized on the TapeStation, and concentration measured on Thermo Fisher Scientific’s Qubit 3 using the DNA HS kit. Libraries were pooled for a QC run on Illumina’s Miseq sequencer running the MiSeq Reagent Nano Kit v2 (300-cycles, 1.4 × 10^6^ reads/run). Data were analyzed using 10X Genomic’s Cell Ranger tool, and cell counts generated were used to rebalance the pool for sequencing on Illumina’s NextSeq 2000, using a P2-100 cycle kit. FASTQ files were generated using Illumina’s BCLConvert. The Cell Ranger (v8.0.1, 10X Genomics) count pipeline was used to align the reads in the FASTQ files to the GENCODE M33 mouse reference transcriptome for calling cells and to generate gene expression counts per cell. This yielded a total of approximately 7800 cells across all experiments.

### scRNA-seq data analysis.

Downstream analysis of mouse scRNA-seq data was performed in R v4.4, using Seurat v4.4. Standard preprocessing quality control was performed and cells with high mitochondrial gene expression and low/high feature counts were filtered out. The SoupX tool corrected for contamination from ambient RNA. To mitigate effects of cell cycle heterogeneity, the difference between the G_2_M and S phase scores was regressed out. We also identified immediate-early genes, calculated their module scores per cell and used it for regression.

Data were normalized using sctransform and all data across experiments were integrated to anchor shared cell populations. Principal components were estimated based on a diagnostic plot (elbow plot) to capture the major structure in the data, which were used to determine UMAP dimensionality reductions (47, 48). Samples from vehicle-treated healthy mouse mammary fat pad and vehicle-treated mammary fat pads opposite tumors were analyzed together to represent NECs.

Following cluster identification, markers were identified for each cluster using the FindAllMarkers function in Seurat. Clusters were annotated for cell types based on published markers, and CellChat (57, 58) was used to analyze changes in interactions between cell types (tumor, immune, pericyte, endothelial, lymphatic). We examined changes in the strength of interactions and probability of interacting ligand-receptor pairs between cell types to identify significant changes in ligand-receptor communication probability. All interactions with *P* values below the resolution limit (*P* = 0.01) for 100 permutations are reported as *P* less than 0.01.

The endothelial cluster was reclustered to identify subtypes and analyzed further across experiments for gene expression. ECs were module scored for early and late G_1_ gene signatures using the top 90 state-specific genes identified from bulk RNA-seq of Fucci2-expressing ECs.

The identified immune cell cluster was reclustered iteratively to identify the macrophage population. Macrophage identity was confirmed by *Cd68* and *Adgre* gene expression and this population was analyzed for M1 and M2 polarization–associated gene expression.

Publicly available human scRNA-seq data from Luo et al. (75) (NCBI GEO accession: GSE262288) were analyzed in R (v4.4) using Seurat (v4.4). Preprocessing was performed, including cell filtering and quality control according to the authors’ methods (75). Data were normalized, integrated, and visualized as described for mouse scRNA-seq. Following clustering, the human EC cluster was identified by *CDH5* expression. Subsetting based on *CDH5* expression and reclustering were repeated until only ECs remained in the Seurat object. Due to low cell numbers, subtypes could not be resolved, and analysis was performed on all ECs from each sample.

### Immune cell analysis.

Tumors from mice with and without CDK4/6i treatment were harvested, minced (3 minutes) in digestion buffer, mechanically dissociated using C-tubes, and enzymatically digested at 37°C (80 rpm, 40 minutes). Spleens were collected in low-glucose DMEM with 5% FBS and centrifuged (314*g*, 5 minutes) and splenocytes were resuspended in red blood cell lysis buffer (Invitrogen, 00-4333-57) for 2 minutes. PBS was added and cells centrifuged (314*g*, 10 minutes) and resuspended in PBS.

Tumor samples were strained, washed, and resuspended in low-glucose DMEM with 5% FBS. Single-cell suspensions were spun (300*g*, 10 minutes), washed, and spun again (300*g,* 5 minutes). Samples were resuspended in 10 mL PBS with 10 mL Lympholyte-M Cell Separation Media (Cedarlane Labs, CL5031) and centrifuged at 1000*g* with low acceleration and no brake. The lymphocyte layer was collected, washed with PBS, pelleted (800*g*, 10 minutes), and resuspended in PBS.

Samples, fluorescence-minus-ones (FMOs; splenocytes), and single-stained and unstained controls were added to a U-bottom plate and spun (1200 rpm, 5 minutes). Cells were resuspended in Live/Dead Fixable Blue Dead Cell Stain (1:800; Thermo Fisher Scientific, L34961) according to the manufacturer’s instructions. Cells were washed and incubated with Mouse Fc Block (1:200; BD Biosciences, 553142) in FACS buffer (2% BSA, 0.08% NaN_3_) with 2% normal mouse serum (NMS) (Valley Biomedical, AS3054) for 15 minutes at 4°C and washed. Samples and FMO stains were made in Brilliant Stain Buffer (BD Biosciences, 563794) with FACS buffer containing 2% NMS and single-stains were made in FACS with 2% NMS. Cells were incubated with antibodies (Supplemental Table 5) for 30 minutes at 4°C and washed with FACS buffer. Cells were fixed, permeabilized, and stained for FOXP3 according to the eBioscience FOXP3/Transcription Factor Staining Buffer Set (Invitrogen, 00-5523-00). Cells were then fixed with 1× FACS lysis buffer (BD Biosciences, 349202) for 10 minutes, resuspended in 120 μL FACS buffer, and analyzed using the Cytek Aurora 5 laser cytometer. Data were analyzed using FCS Express (https://denovosoftware.com/) and FlowJo.

### Statistics.

For all analyses, a *P* value of 0.05 or less was considered significant and a *P* value of greater than 0.05 non-significant (NS). Continuous variables were graphed as mean ± SEM with data shown as dots. Violin plots show the median as a black line. For comparisons between 2 groups, an unpaired nonparametric Mann-Whitney test was used. Fucci2 quantifications in healthy and tumor tissues were compared using the Wilcoxon matched-pairs signed-rank test, as each mouse had both tissues analyzed. Two-way ANOVA and Šídák’s multiple-comparison test were used to compare the means between 3 or more groups when 2 independent variables were present. The Kruskal-Wallis test followed by the Wilcoxon rank-sum test with post hoc Holm correction was used to calculate *P* values for pairwise comparisons in the scRNA-seq data, including module scores and gene expression. Tumor measurements over time were compared using the repeated-measures 2-way ANOVA and Šídák’s multiple-comparison test. Simple linear regression analysis was determined the relationship between hypoxia or perfusion and tumor volume. Differentially expressed genes between TECs+vehicle and TECs+CDK4/6i were identified with Seurat’s Findmarkers function that uses the Wilcoxon rank-sum test. All graphs and statistics were generated using GraphPad Prism (v10.5) or ggplot2 and ggpubr packages in R (v4.4).

### Animal study approval.

All animal procedures used were approved by the UVA Animal Care and Use Committee (protocol 4277) and satisfied all ethical regulations. Animals were housed within a barrier facility with approved facilities for animal care, staffed with trained animal care technicians and a full-time veterinary staff.

### Data availability.

Raw and processed files for the bulk RNA-seq and scRNA-seq are publicly available and uploaded to the NCBI GEO with accession numbers GSE313493 and GSE313369, respectively. Values for all data points in graphs are reported in the Supporting Data Values file. All other data are available upon request.

## Author contributions

SRC, KKH, ACD, and RJP designed studies. SRC, VMM, AC, JWA, MGJ, ZM, and MRS conducted experiments. Data were acquired by SRC and analyzed by SRC, NG, GG, UP, NWC, ACD, and RJP provided reagents. ACD provided D4M-3A cells. SRC, GG, NG, and KKH wrote the manuscript.

## Conflict of interest

The authors have declared that no conflict of interest exists.

## Funding support

This work is the result of NIH funding, in whole or in part, and is subject to the NIH Public Access Policy. Through acceptance of this federal funding, the NIH has been given a right to make the work publicly available in PubMed Central.

- NIH grants R01HL146056 and R01HL171284 (to KKH).
- NIH grants 2R01CA177875 and R01CA2558451 (to ACD).
- NIH grants R01CA279134 and R21CA286367 (to RJP).
- American Heart Association grants CDA1051175 (to NG) and CDA938744 (to GG).
- NIH grants T32HL007284 (to SRC, JWA, and MGJ), T32CA009109 (to MRS), F31CA288057 (to SRC), and F31HL172647 (to JWA).
- UVA Precision Immunomedicine grant DN001637 (to VMM).
- American Heart Association Predoctoral Fellowships 25PRE1362748 (to VMM) and 23PRE1026720 (to AC).
- Robert R. Wagner Fellowship (to AC and ZM).
- National Science Foundation Graduate Research Fellowship (to MRS).
- National Cancer Institute grant P30CA044579 (to the UVA Cancer Center, Flow Cytometry Core Facility, and internal pilot project award to KKH, ACW, and SRC).

## Supplementary Material

Supplemental data

Supplemental table 1

Supporting data values

## Figures and Tables

**Figure 1:**
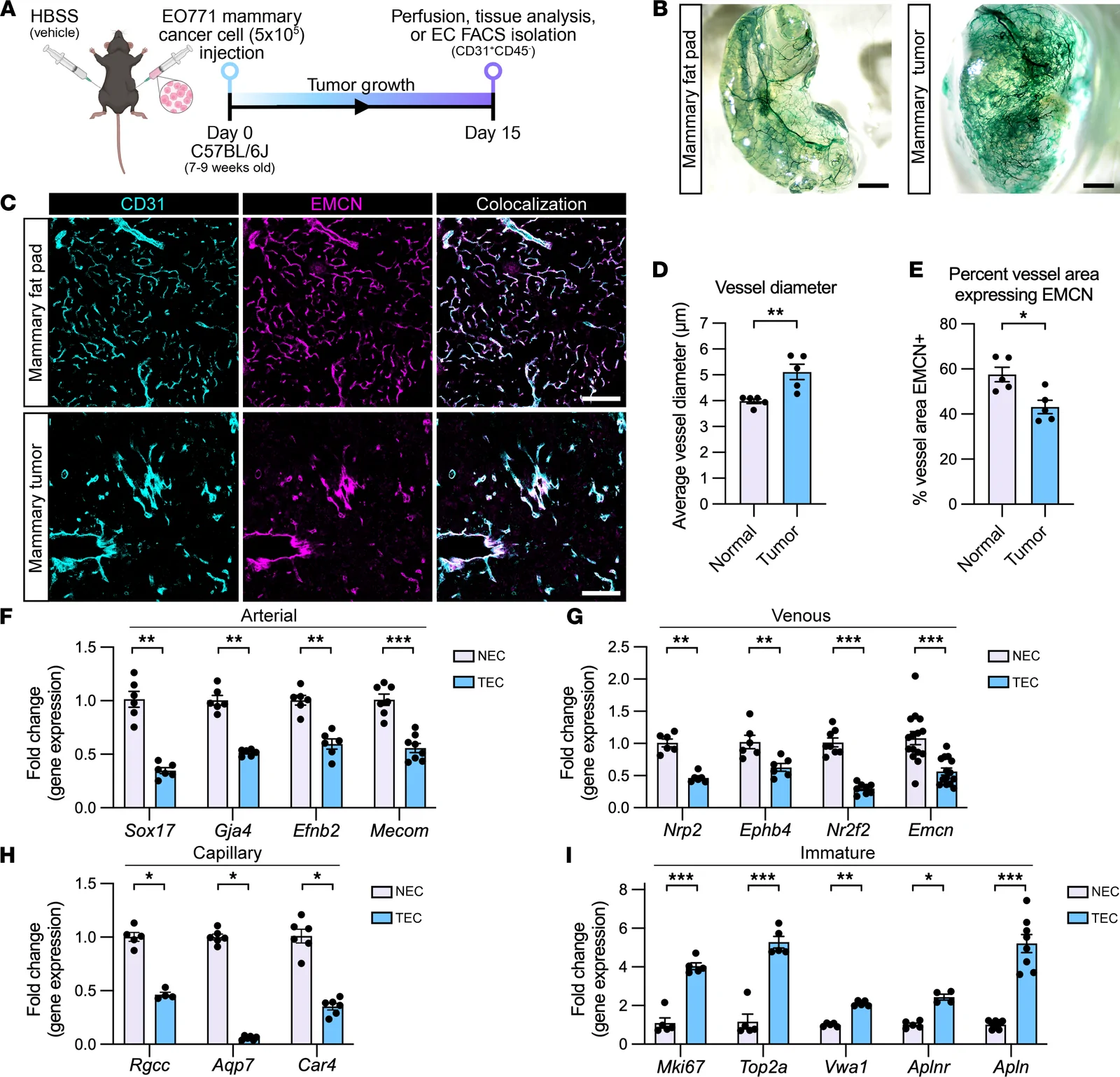
Tumor vessels are phenotypically immature and tumor endothelial cell (TEC) specification gene expression is reduced. (**A**) Murine mammary cancer (EO771) injection and experimental timeline. (**B**) Bright-field images of green Vascupaint–perfused and cleared mammary fat pad and tumor. (**C**) Representative confocal images of mammary fat pad and tumor stained for CD31 (vessels) and EMCN (venous-enriched). (**D**) Vessel diameter quantification (*n* = 5 mice/group). (**E**) Percentage vessel area (CD31^+^) colocalized with EMCN. (**F**–**I**) qPCR analysis of arterial- (**F**), venous- (**G**), capillary- (**H**) and immaturity-enriched gene (**I**) expression in normal mammary endothelial cells (NECs) and TECs (2 independent experiments, up to 15 mice/group). *P* values were calculated using an unpaired Mann-Whitney test (**C**–**I**). All data presented as mean ± SEM. *P < 0.05, **P < 0.01, ***P < 0.001. NS, nonsignificant. Scale bars: 1 mm (**B**) and 100 μm (**C**).

**Figure 2:**
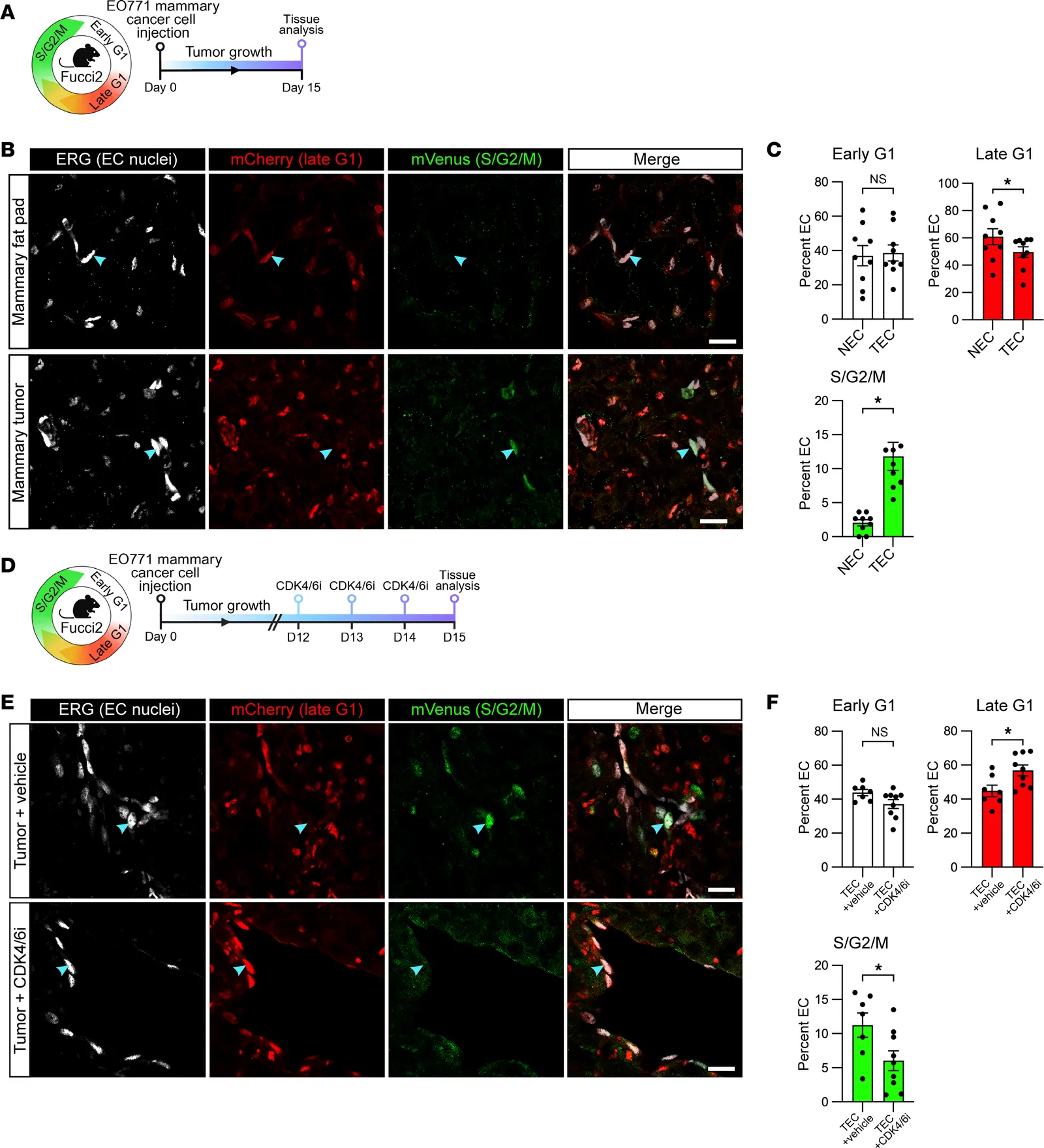
TECs have decreased late G_1_ and increased S/G_2_/M proportions compared with NECs, and CDK4/6i promotes late G_1_ states. (**A**) Experimental timeline for the EO771 model in global Fucci2-expressing mice. (**B**) Representative confocal images of NEC and TEC nuclei (ERG^+^) from global Fucci2-expressing mice with cell cycle states indicated by ERG^+^ only (early G_1_), ERG^+^mCherry^+^ (late G_1_), and ERG^+^mVenus^+^ (S/G_2_/M). Blue arrowheads: late G_1_ (top panels) and S/G_2_/M (bottom panels) EC nuclei. (**C**) Fucci2 expression quantification (2 independent experiments, 55–75 ECs averaged/mouse, *n* = 9 mice/group). (**D**) Experimental timeline for the EO771 model with CDK4/6i treatment in Fucci2 mice. (**E**) Representative confocal images of tumors from vehicle- or CDK4/6i-treated Fucci2 mice. Blue arrowheads: S/G_2_/M (top panels) and late G_1_ (bottom panels) EC nuclei. (**F**) Quantification of Fucci2 TEC cycle states (2 independent experiments, 200–400 ECs averaged/mouse, *n* = 7–9 mice/group). *P* values calculated using the Wilcoxon matched-pairs signed-rank test (**C**) or unpaired Mann-Whitney test (**F**). *P < 0.05. Scale bars: 20 μm (**B** and **E**).

**Figure 3:**
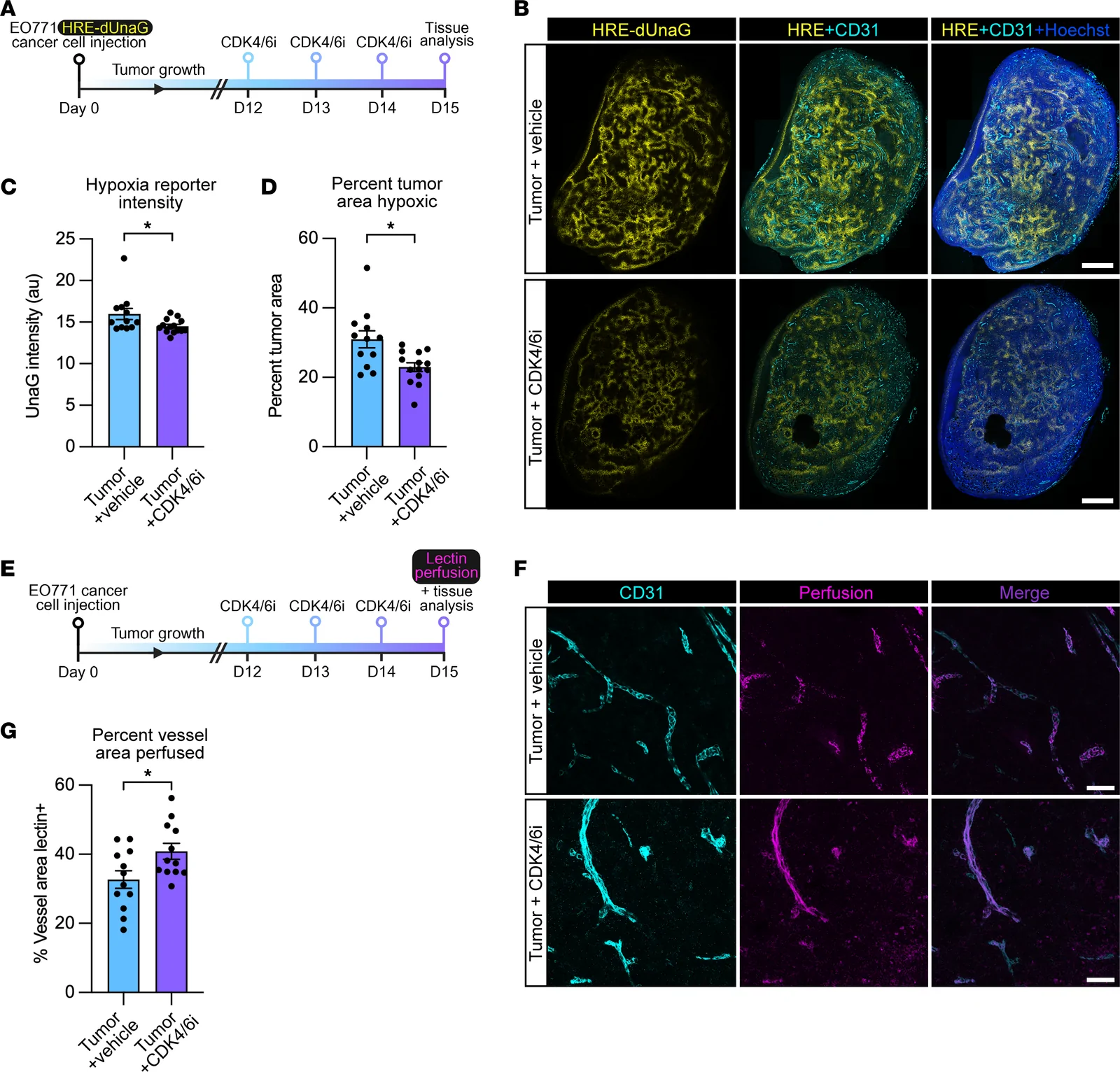
Hypoxic signaling and vessel perfusion in CDK4/6i-treated tumors. (**A**) Experimental timeline for the EO771 HRE-dUnaG hypoxia reporter model. (**B**) Representative confocal images of EO771 tumors with and without CDK4/6i treatment expressing HRE-dUnaG (yellow) and stained for CD31 (cyan) and with Hoechst. (**C**) HRE reporter intensity and (**D**) percentage hypoxic tumor area based on dUnaG fluorescence in tumor sections (2 independent experiments, *n* = 12–14 mice/group). (**E**) Experimental timeline for lectin-FITC perfusion 20 minutes before EO771 tumor harvest. (**F**) Representative confocal images of tumor sections from vehicle- or CDK4/6i-treated mice perfused with lectin (magenta) and stained for CD31 (cyan). (**G**) Vessel perfusion quantified by average percentage area of vessels (CD31^+^) positive for lectin fluorescence (2 independent experiments, *n* = 12 mice/group). *P* values calculated using unpaired Mann-Whitney test (**C**, **D**, and **G**). *P < 0.05. Scale bars: 1 mm (**B**) and 50 μm (**F**).

**Figure 4:**
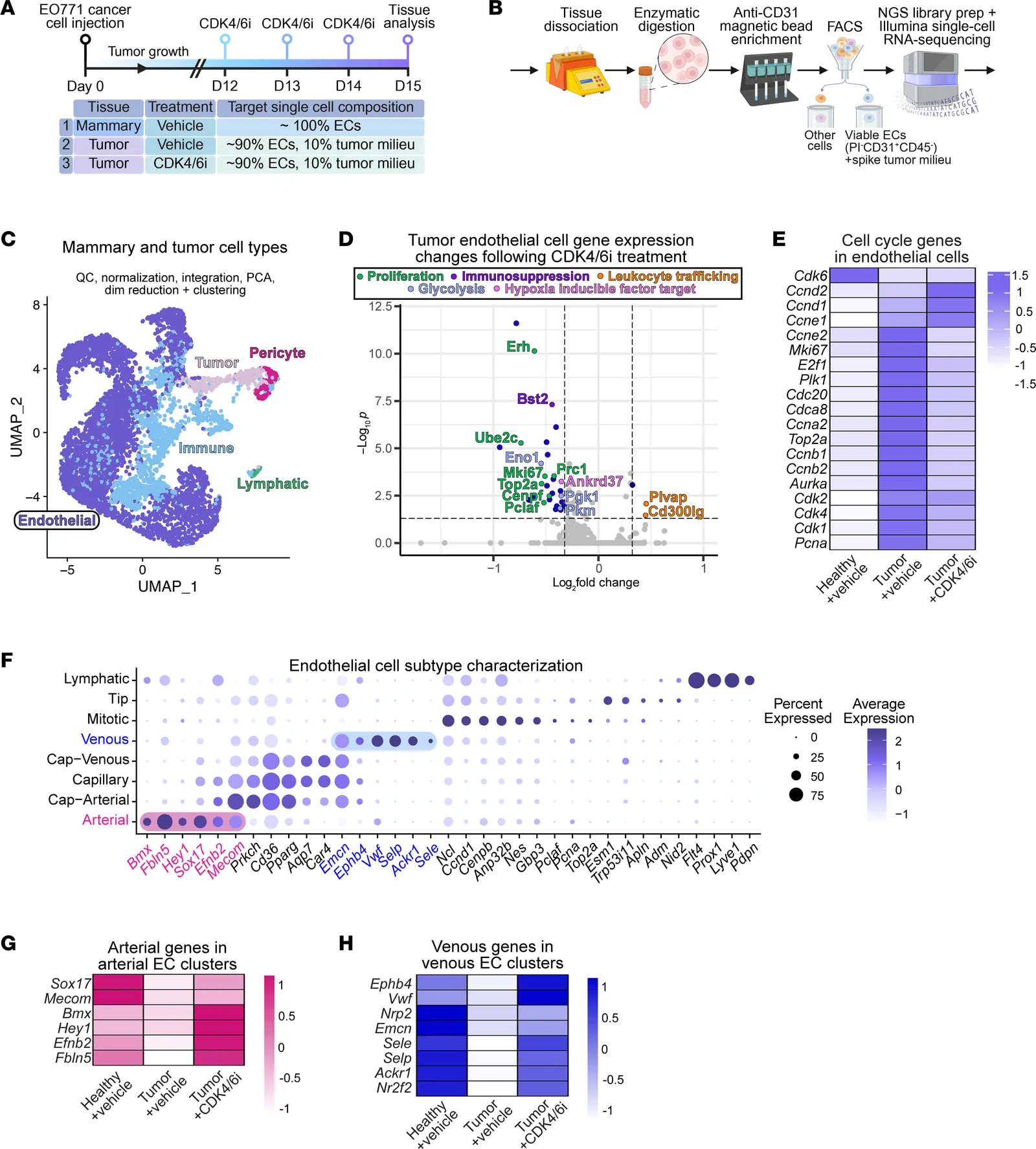
Mammary, tumor, and CDK4/6i-treated tumor EC transcriptomic changes include genes associated with proliferation and specification. (**A**) Experimental schematic for scRNA-seq of mammary fat pads, tumors, and CDK4/6i-treated tumors pooled in 2 independent experiments. (**B**) Workflow for isolating viable ECs and sequencing analysis. Cells from initial tumor single-cell suspensions were added to TEC samples for analysis of cell-type specific interactions. (**C**) UMAP of integrated datasets annotated by cell type. (**D**) Volcano plot of differentially expressed genes between ECs from vehicle- versus CDK4/6i-treated tumors calculated from scRNA-seq data. Colored dots represent *P* < 0.05 and |fold change| > 1.25, gray dots represent *P* > 0.05, |fold change| < 1.25, or both. Label colors indicate genes associated with proliferation (green), immunosuppression (purple), glycolysis (light blue), hypoxia-inducible factor (HIF) target, and leukocyte trafficking (orange). (**E**) Scaled heatmap of relative proliferation-associated gene expression in ECs from healthy mammary+vehicle, tumor+vehicle, and tumor+CDK4/6i. (**F**) Dot plot of EC subtype annotation and gene expression, including arterial (pink) and venous (blue). (**G** and **H**) Scaled heatmaps of relative arterial and venous gene expression in arterial and venous clusters, respectively.

**Figure 5:**
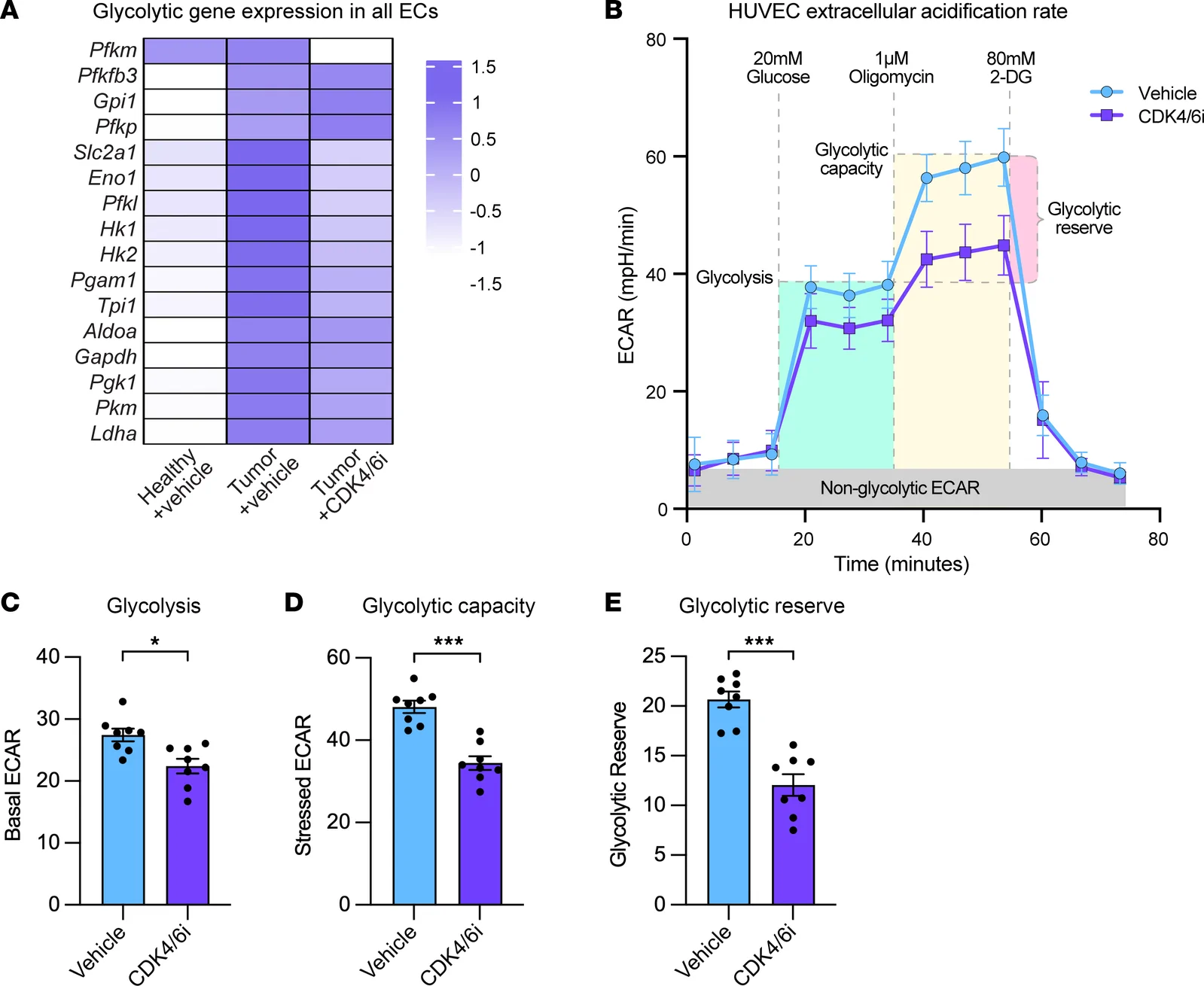
CDK4/6i alters TEC metabolic gene expression and inhibits EC glycolytic activity. (**A**) Heatmap of average expression of glycolytic genes in ECs from healthy mammary+vehicle, tumor+vehicle, and tumor+CDK4/6i scRNA-seq. (**B**) Representative HUVEC extracellular acidification rate (ECAR) traces following 24 hours of CDK4/6i pretreatment (*n* = 8/condition), during the glycolytic stress test. Injection points indicate glucose (20 mM), oligomycin (1 μM; ATP synthase inhibitor), and 2-deoxy-D-glucose (80 mM; glycolysis inhibitor). Glycolysis (green), glycolytic capacity (yellow), and glycolytic reserve (pink) are labeled. (**C**) Basal glycolysis measured as change in ECAR from baseline after glucose addition. (**D**) Glycolytic capacity measured as difference between ECAR after oligomycin addition and baseline. (**E**) Glycolytic reserve, the difference in ECAR from basal glycolysis to glycolytic capacity. *P* values were calculated with unpaired Mann-Whitney tests (**C**–**E**). *P < 0.05, ***P < 0.001.

**Figure 6:**
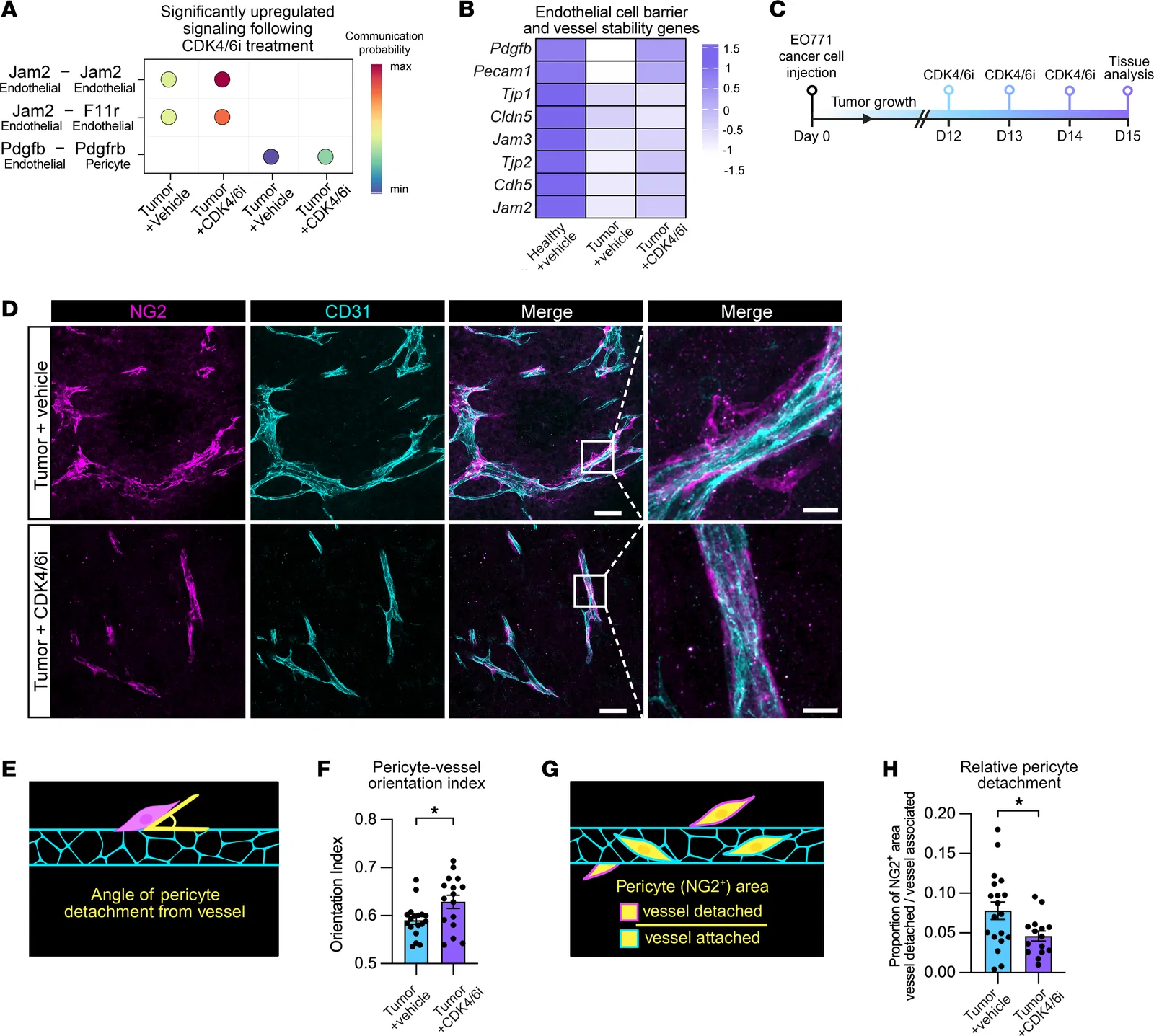
CDK4/6i enhances expression of genes associated with cellular junctions and pericyte communication in TECs. (**A**) CellChat (57, 58) bubble plot showing significantly increased (*P* < 0.01) ligand-receptor communication probability between TECs and TECs or pericytes following CDK4/6i. (**B**) Heatmap of relative EC expression of genes regulating pericyte recruitment and adherens and tight junctions in mammary+vehicle, tumor+vehicle, and tumor+CDK4/6i. (**C**) Experimental timeline for EO771 model with CDK4/6i treatment. (**D**) Representative confocal images of vehicle- and CDK4/6i-treated tumors stained for pericytes (NG2, magenta), vessels (CD31, cyan), and with Hoechst (scale bars: 50 μm). White boxes indicate areas magnified in right panels (scale bars: 10 μm). (**E**) Schematic illustrating the angle (yellow) of pericyte (magenta) detachment from vessels (cyan) measurement. (**F**) Orientation index calculated using the *circ std* function in Matlab using average angle of pericyte detachment (radians) per tumor (2–5 images; 4 replicate experiments, *n* = 16–18 mice/group). Higher values represent improved pericyte-vessel association. (**G**) Schematic illustrating measurements used to calculate relative pericyte detachment. (**H**) Relative pericyte detachment calculated as the ratio of NG2^+^ vessel–detached to NG2^+^ vessel–attached area and averaged per tumor (4 replicate experiments, *n* = 15–19/group). *P* values calculated with unpaired Mann-Whitney test (**F** and **H**). *P < 0.05.

**Figure 7:**
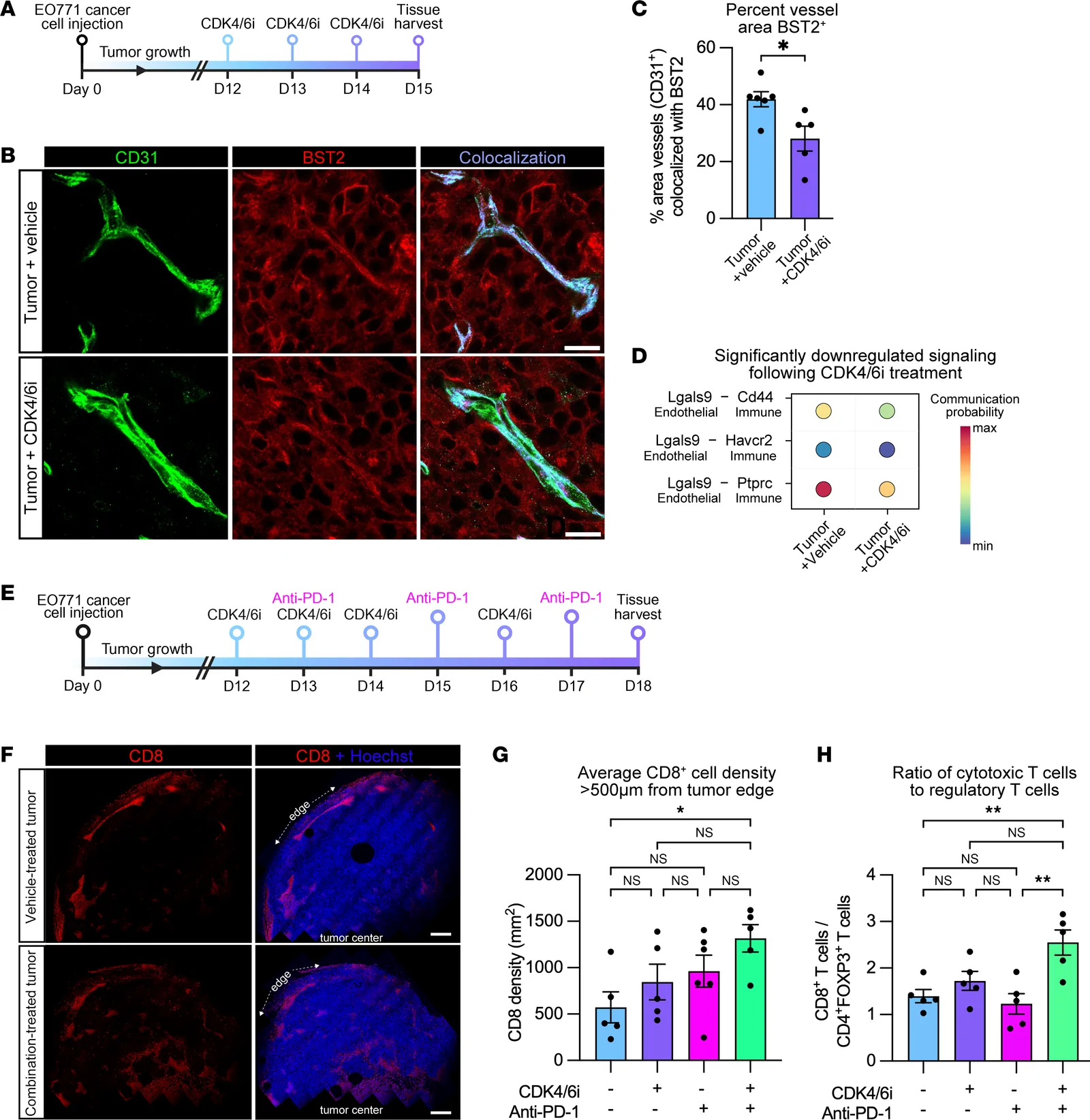
CDK4/6i alters tumor EC immune signaling and cooperates with immunotherapy. (**A**) Experimental timeline for EO771 model with CDK4/6i treatment. (**B**) Representative confocal images of tumors with and without CDK4/6i immunostained for CD31 (green) and BST2 (red) with colocalization (blue). (**C**) Average percentage vessel (CD31^+^) area colocalized with BST2 (4–7 ROI/tumor, *n* = 5–6 tumors/group). (**D**) Significantly decreased (*P* < 0.01) ligand-receptor communication probability between TECs and immune cells following CDK4/6i. (**E**) Experimental design for EO771 model with groups receiving CDK4/6i (150 mg/kg/day), anti–PD-1 (15 mg/kg), combination therapy, or vehicle and IgG2a isotype control. (**F**) Representative confocal images of tumor sections immunostained for CD8 (red) and with Hoechst (blue) following vehicle (top) or combination (bottom) treatment. Images span the tumor edge to center. (**G**) CD8^+^ density greater than 500 mm from tumor edge (*n* = 5–6 mice/group). (**H**) Ratio of CD8^+^ to CD4^+^FOXP3^+^ T cells in tumors quantified by flow cytometry (*n* = 5 mice/group). *P* values calculated with unpaired Mann-Whitney test (**C**) and 2-way ANOVA with Šídák’s multiple-comparison test (**G** and **H**). Scale bars: 20 mm (**B**) and 500 mm (**F**). *P < 0.05; **P < 0.01.

**Figure 8:**
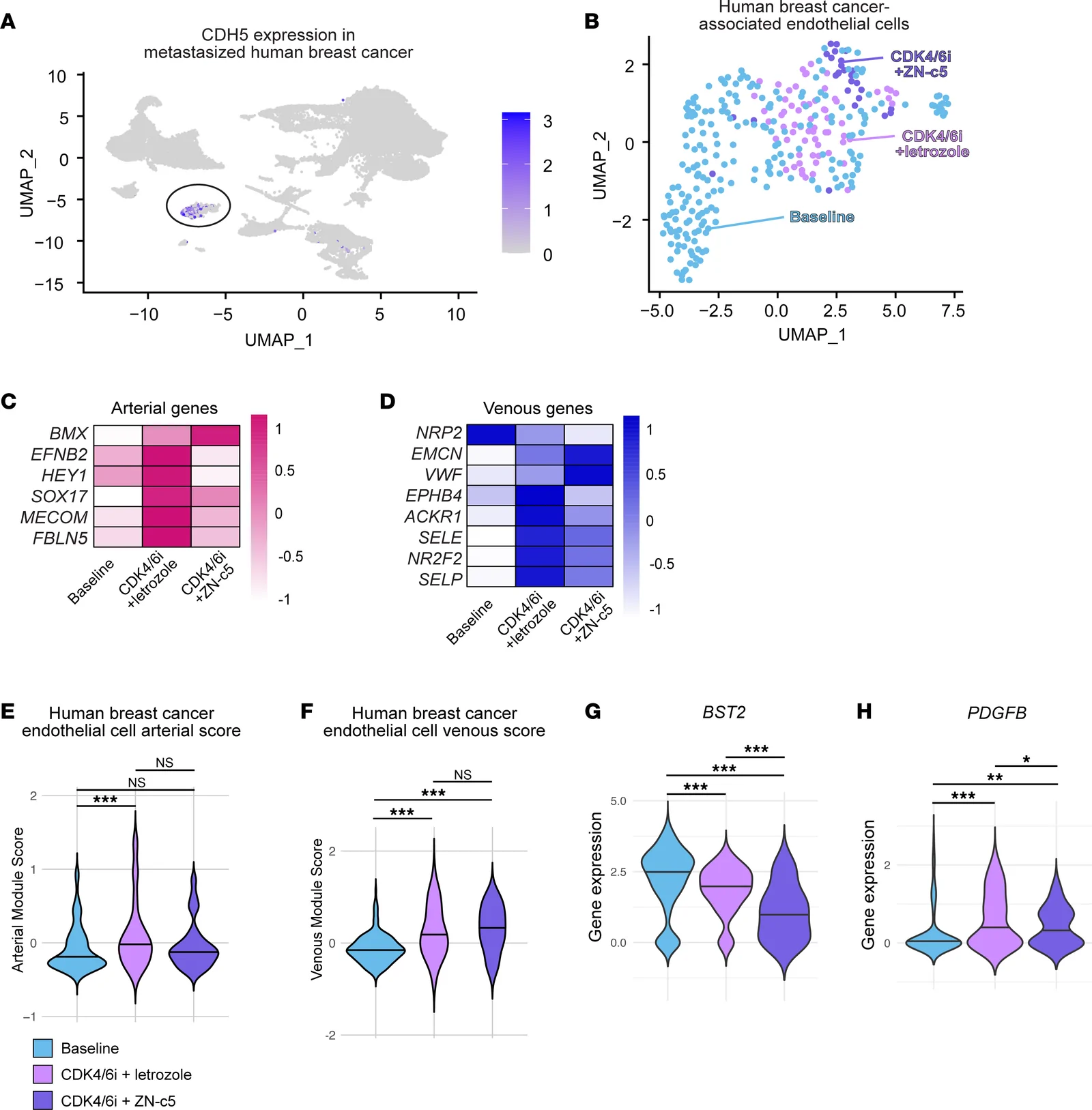
Human TEC scRNA-seq reveals signaling changes and enhanced specification. (**A**) scRNA-seq of human HR^+^HER2^–^ breast cancer liver metastasis biopsies (GEO GSE262288) SCT-integrated and visualized with UMAP using Seurat. ECs identified by CDH5 expression. (**B**) ECs subset from **A** and annotated by sample identity: baseline (*n* = 5, 228 ECs), CDK4/6i+letrozole (*n* = 1, 66 ECs), and CDK4/6i+ZN-c5 (*n* = 1, 27 ECs). Scaled heatmap of relative arterial (**C**) and venous (**D**) gene expression in human TECs split by condition. Violin plots of TECs split by conditions and module scored for arterial (**E**) and venous (**F**) gene expression using respective genes in **C** and **D**. Violin plots of TEC *BST2* (**G**) and *PDGFB* (**H**) expression split by condition. *P* values (**E**–**H**) calculated using Kruskal-Wallis tests followed by pairwise Wilcoxon rank-sum tests with post hoc Holm correction. Violin plots show median as a black line. *P < 0.05; **P < 0.01; ***P < 0.001.

## Version 1 09/10/2026

In-Press Preview

## Version 2 10/22/2026

Electronic publication
