# Supplementary material for: Endothelial cell cycle inhibition enables blood vessel maturation to normalize the tumor vasculature
Source: JCI Insight. 2026 Sep 10;11(20):e203732. doi: 10.1172/jci.insight.203732 (PMC13726565; doi:10.1172/jci.insight.203732)
Supplement: Supplementary file 1 [file jciinsight-11-203732-s102.pdf]

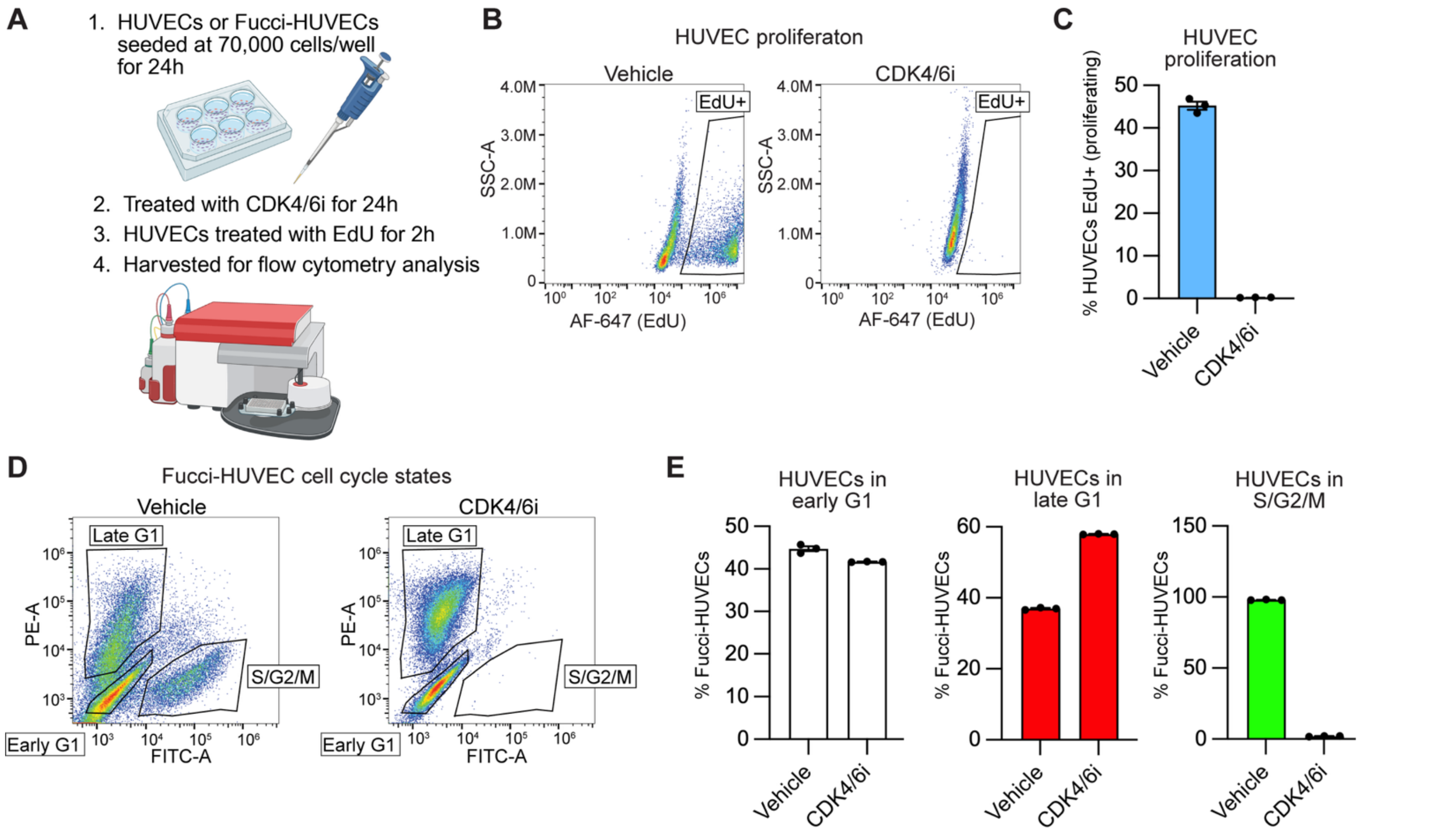

Supplemental Figure 1: Palbociclib effects on endothelial cell cycle in vitro. (**A**) Experimental design for HUVEC proliferation assays (B-E) with vehicle (DMSO) or CDK4/6i treatment for 24 hours, created in BioRender. Cain, S. (2026) https://BioRender.com/b4hosai. (**B**) Flow cytometric analysis for proliferative (EdU^{+}) HUVECs treated with vehicle (DMSO) or CDK4/6i (1μM). EdU (5μM) incorporated for 2 hours prior to analysis. (**C**) Quantification of flow cytometry gating for HUVEC proliferation (EdU+) (n=3). (**D**) Flow cytometric analysis of Fucci-HUVECs treated with vehicle (DMSO) or CDK4/6i (1.5μM). (**E**) Quantification of flow cytometry gating for cell cycle states in Fucci-HUVECs (n=3). All data are expressed as mean ± standard error of the mean (SEM).

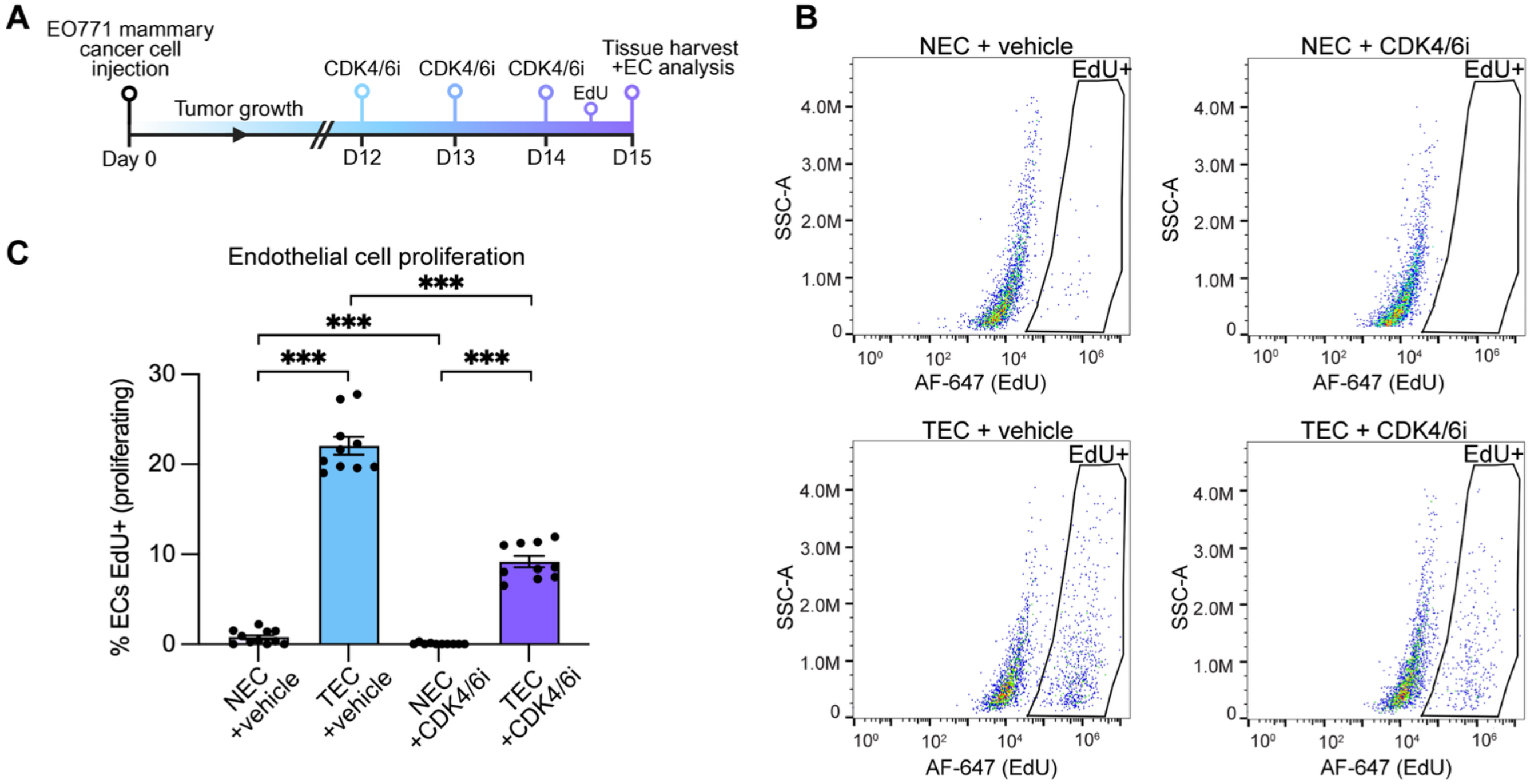

Supplemental Figure 2: Palbociclib reduces TEC proliferation in vivo. (**A**) Experimental timeline for mouse mammary cancer model with CDK4/6i treatment and EdU injections prior to harvest, created in BioRender. Cain, S. (2026) https://BioRender.com/pyb6q3s. (**B**) Representative flow cytometry analysis of EdU^{+} (Alexa Fluor 647) EC (CD31^{+}CD45^{-}) from healthy mammary fat pad and tumors treated with CDK4/6i or vehicle (10% DMSO). (**C**) Quantification of EC proliferation based on EdU^{+} gating in (B) (performed in triplicate, n=10-11). *P*-values calculated with Prism version 10.5.1 using two-way ANOVA and Šídák's multiple comparisons test (C). All data are expressed as mean$\pm$SEM; ****p*<0.001, ns=nonsignificant.

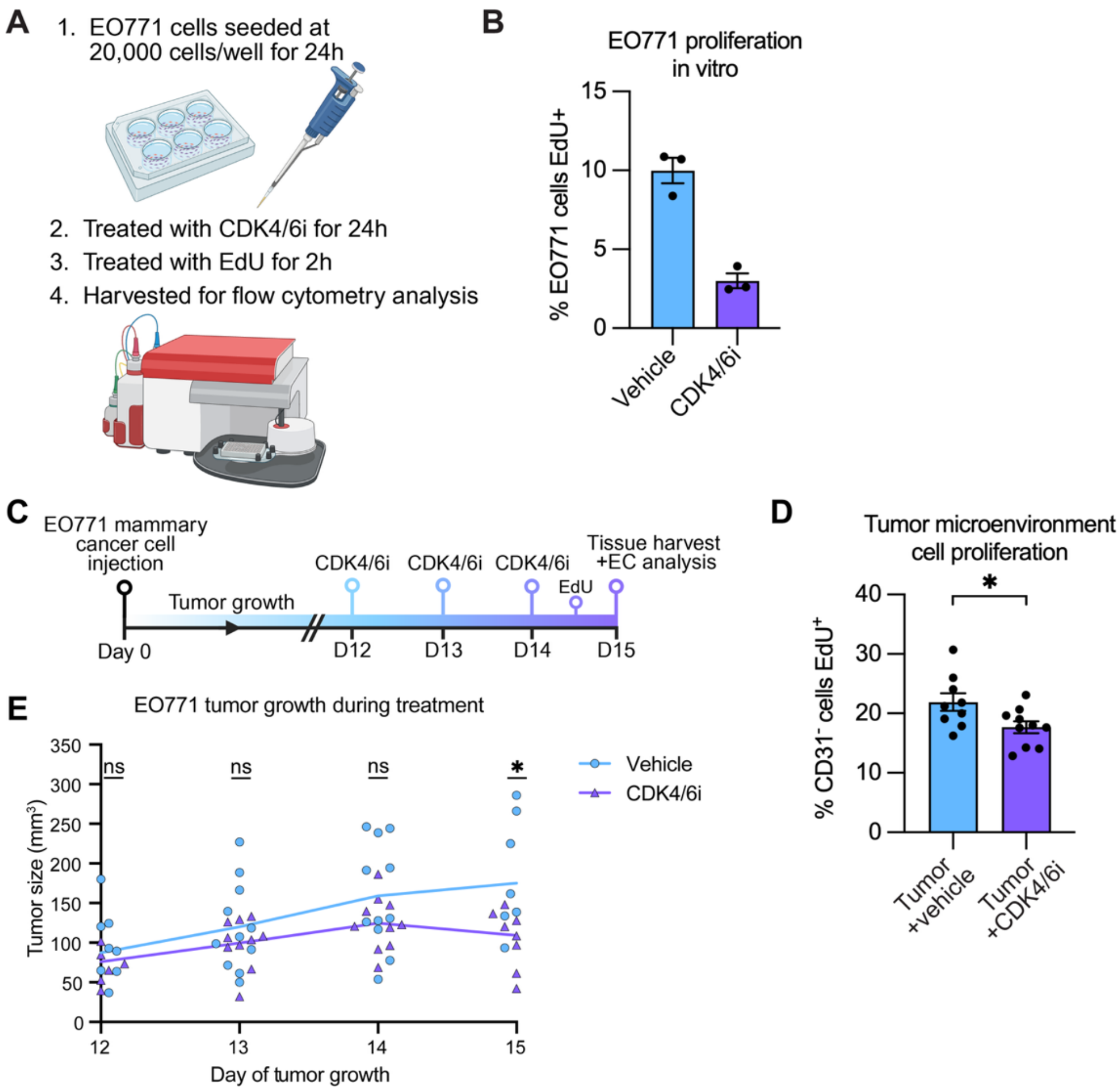

Supplemental Figure 3: Palbociclib effects on cell proliferation and tumor growth. (A) Experimental design for EO771 proliferation assay in vitro with vehicle (DMSO) or CDK4/6i treatment for 24 hours and EdU (5 μM) incorporation 2 hours prior to analysis. (**B**) Quantification of flow cytometry for proliferative (EdU^{+}) EO771 cells in culture after vehicle (DMSO) or CDK4/6i (10μM) treatment for 24h (n=3). (**C**) Experimental timeline for mouse mammary cancer model with CDK4/6i treatment and EdU injections prior to harvest. (**D**) CD31- tumor cell (CD31 column flow through) proliferation in vivo analyzed with flow cytometry (performed in triplicate, n=9-10 mice total). (**E**) Tumor growth of EO771 (two experiments, n=10-11 total). *P*-values calculated with Prism version 10.5.1 unpaired nonparametric Mann-Whitney statistical test (D) and repeated measures two-way ANOVA and Šídák's multiple comparisons test (E). All data are expressed as mean±SEM; **p*<0.05, ns=nonsignificant. (A) and (C) created in BioRender; Cain, S. (2026) https://BioRender.com/emejn1r.

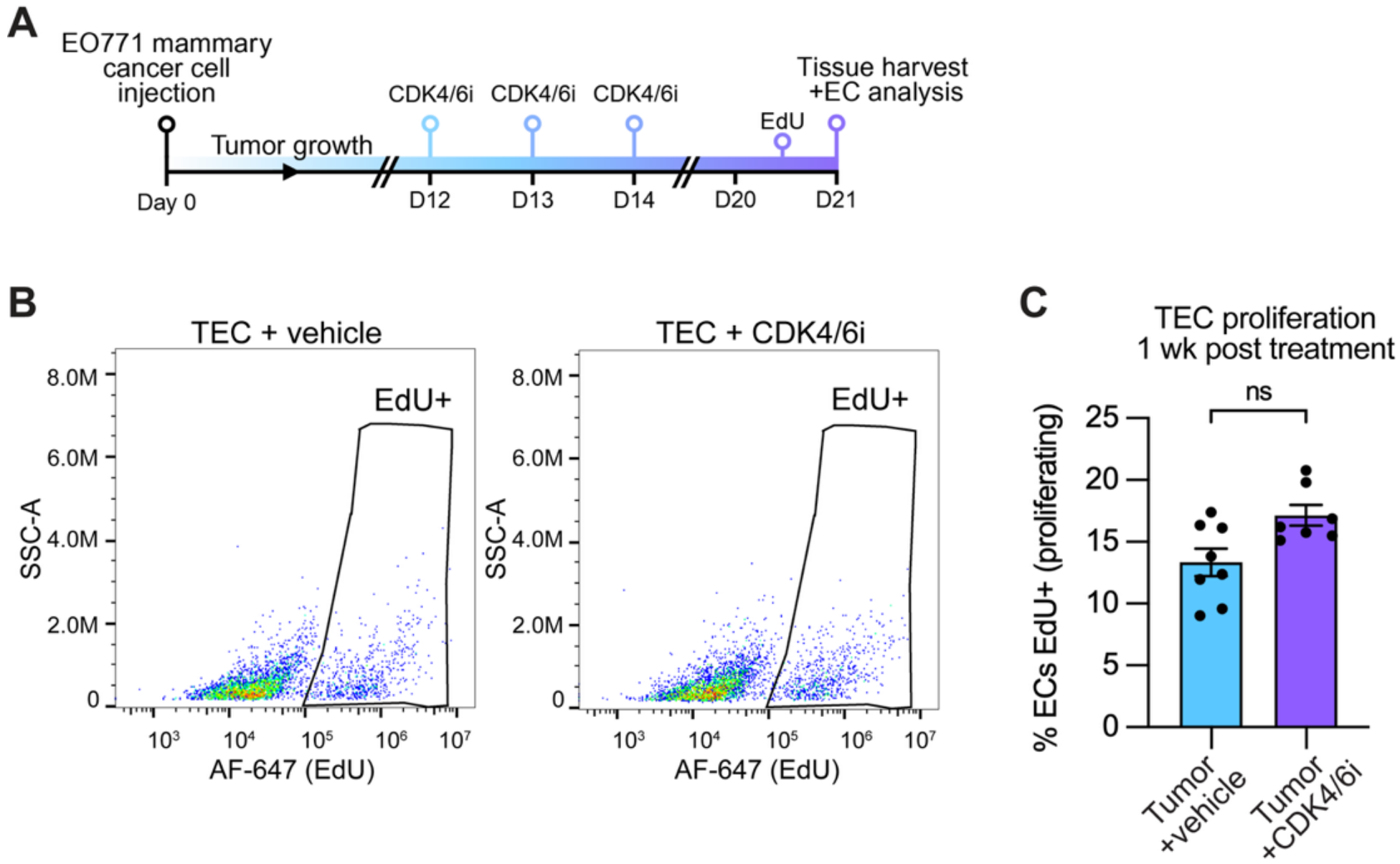

Supplemental Figure 4: TEC proliferation following cessation of Palbociclib treatment. (**A**) Experimental timeline for mouse mammary cancer model with CDK4/6i treatment ending one week prior to EdU injection and harvest, created in BioRender. Cain, S. (2026) https://BioRender.com/apbgso1. (**B**) Representative flow cytometry analysis of EdU^+ (Alexa Fluor 647) EC ($CD31^+CD45^-$) from tumors treated with CDK4/6i or vehicle (10% DMSO). (**C**) Quantification of TEC proliferation based on EdU^+ gating in (B) (n=7-8). *P*-values calculated with Prism version 10.5.1 unpaired nonparametric Mann-Whitney statistical test (C). All data are expressed as mean±SEM; ns=nonsignificant.

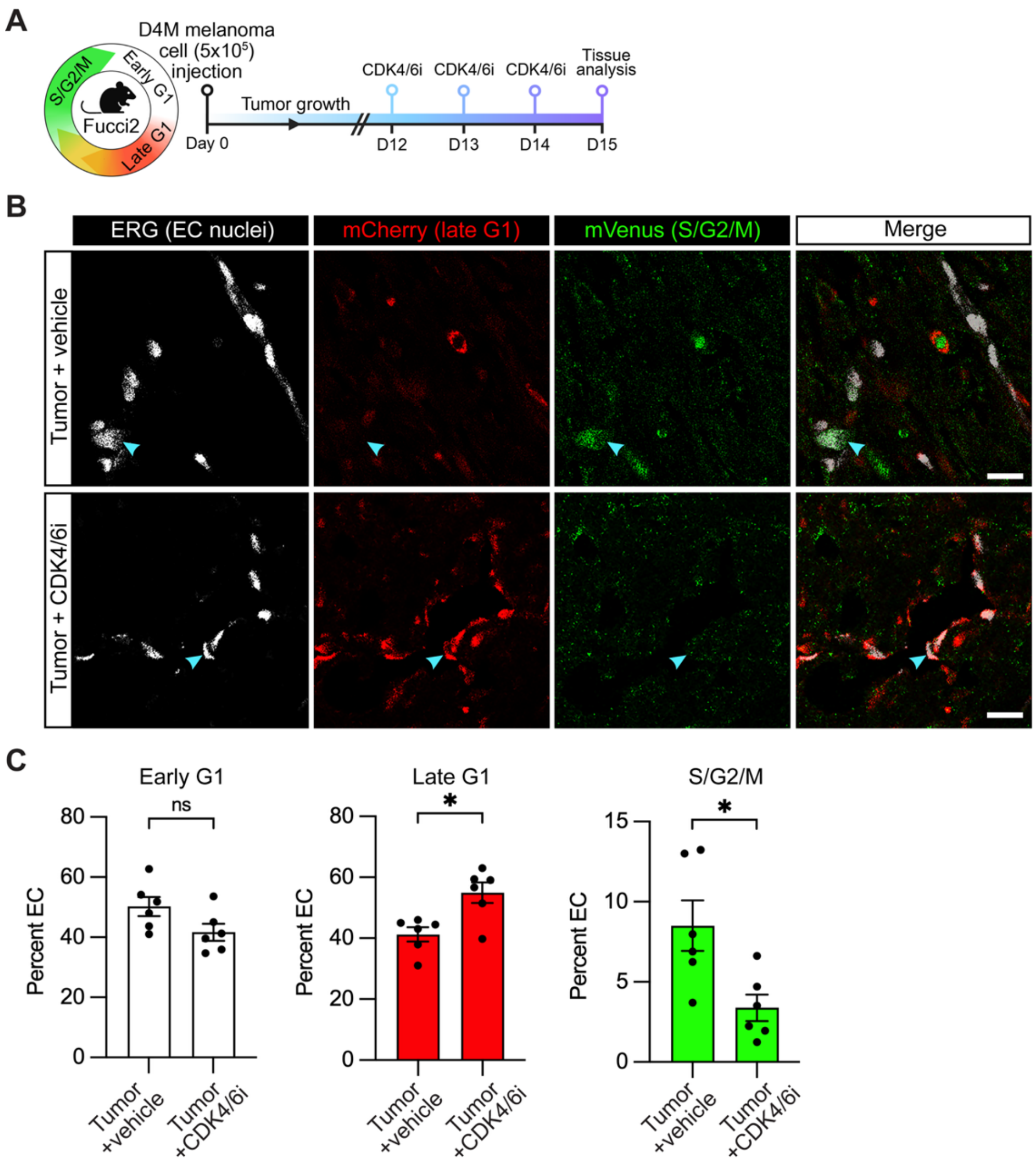

Supplemental Figure 5: Palbociclib decreases proliferation and promotes late G1 state in D4M melanoma TECs. (**A**) Experimental timeline for mouse D4M melanoma model with CDK4/6 treatment in global Fucci2-expressing mice, created in BioRender. Cain, S. (2026) https://BioRender.com/39ztfeh. (**B**) Representative confocal images from tumors in Fucci2 mice treated with CDK4/6i or vehicle. Blue arrowheads identify EC nuclei that are in S/G2/M (top panels) and late G1 (bottom panels). Scale bar = 20µm. (**C**) Quantification of Fucci2 EC cycle states (experimental replicates, 400 ECs/mouse, n=6 mice per group). Unpaired nonparametric Mann-Whitney statistical test. All data are expressed as mean±SEM; *p<0.05, ns=nonsignificant.

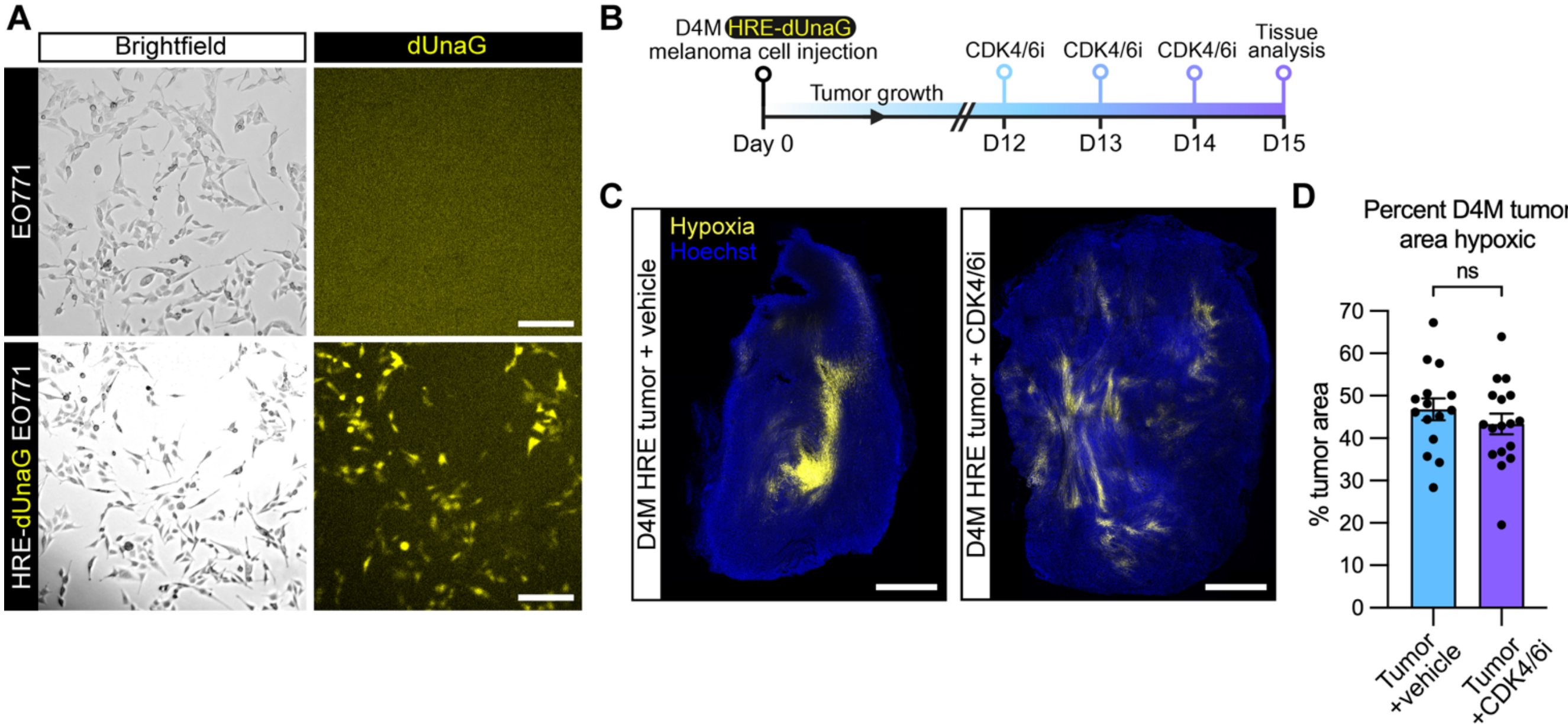

Supplemental Figure 6: HRE expression in EO771 cells and D4M tumors. A) Validation of HRE-dUnag reporter EO771 transduction. EO771 cells grown in media for 36 hours supplemented with puromycin (1µg/mL) and CoCl2 (50µM) imaged with brightfield (left) or fluorescent (right) microscopy. **(B)** Experimental timeline for mouse D4M tumor model expressing a hypoxia reporter, HRE-dUnaG, created in BioRender. Cain, S. (2026) https://BioRender.com/3asknhs. (**C**) Representative confocal images of vehicle- and CDK4/6i-treated D4M tumors expressing the HRE-dUnaG HIF activity reporter (yellow) and immunostained for CD31 (endothelial cells) and Hoechst. (**D**) Quantification of percent area tumor hypoxic based on dUnaG fluorescence in tumor sections (replicate experiments, n=15-17 mice per group). Unpaired nonparametric Mann-Whitney statistical test. All data are expressed as mean±SEM; ns = not statistically significant. Scale bars = 200µm (A) = 1mm (C).

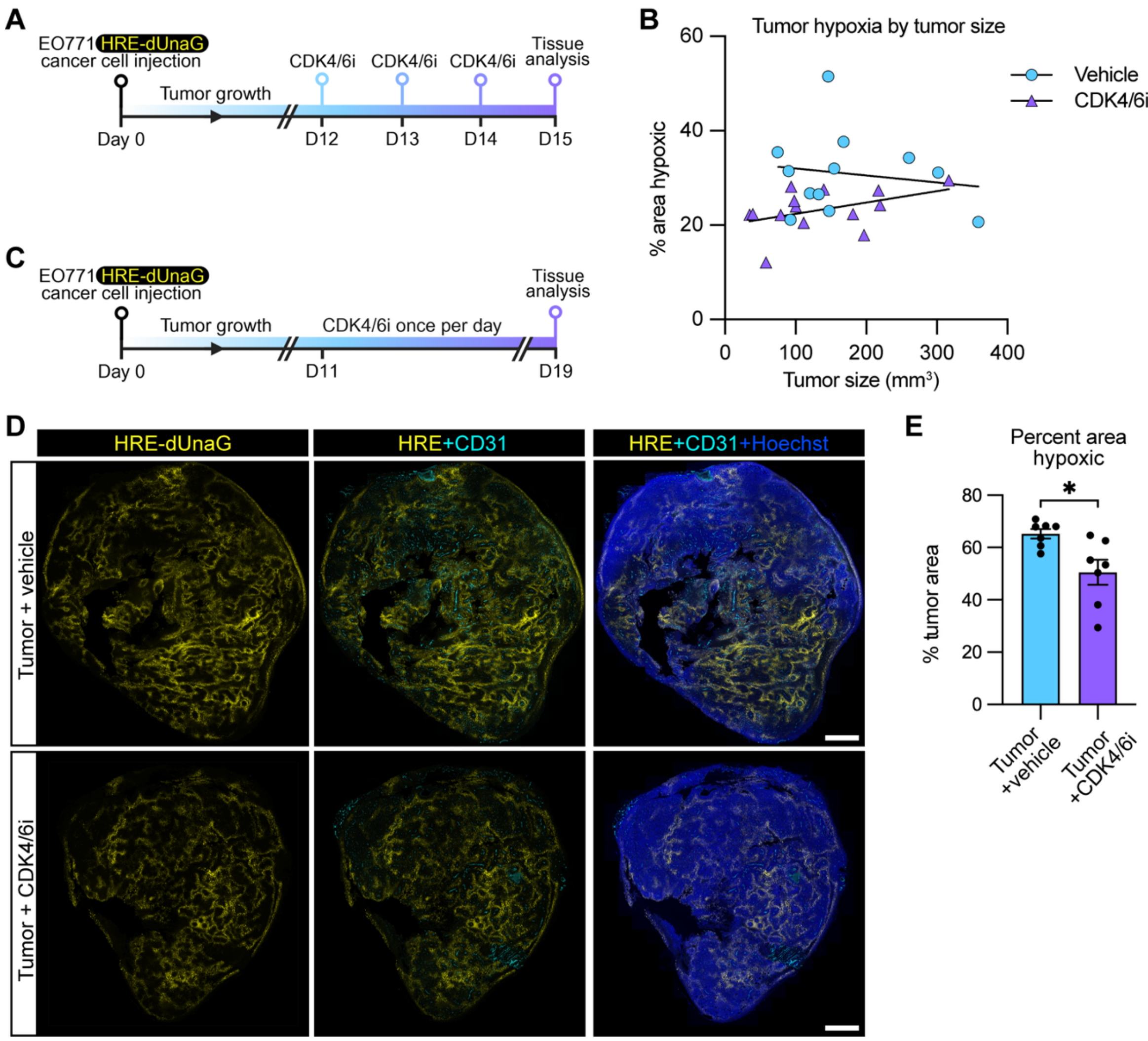

Supplemental Figure 7: **Palbociclib reduces hypoxia response element (HRE) expression across a range of EO771 tumor sizes, and reduced hypoxia is maintained with extended treatment.** (**B**) Experimental timeline for EO771 mouse mammary cancer with hypoxia reporter, HRE-dUnaG, and CDK4/6i treatment at 150mg/kg/day for 3 days. (**C**) Linear regression analysis of tumor volume (mm^3) vs. percent area hypoxic. Vehicle R squared = 0.024 and CDK4/6i treated R squared = 0.187. Line slopes not significantly different (p=0.2425), line elevation is significantly different (p=0.0111). (**D**) Experimental timeline for EO771 mouse mammary cancer with hypoxia reporter, HRE-dUnaG, and CDK4/6i treatment at 150mg/kg/day for 8 days. Scale bars = 1mm. (**E**) Representative confocal images of vehicle- or CDK4/6i-treated EO771 tumors expressing reporter for HIF activity (HRE-dUnaG, yellow) and stained for CD31 (vessels) and Hoechst. (**F**) Quantification of percent tumor hypoxia based on dUnaG fluorescence in tumor sections (n=7-8 mice per group). Statistics calculated with Prism version 10.5.1 simple linear regression (B) and unpaired nonparametric Mann-Whitney statistical test (E) with data expressed as mean±SEM; *p<0.05. (A) and (C) created in BioRender; Cain, S. (2026) https://BioRender.com/988vxtl.

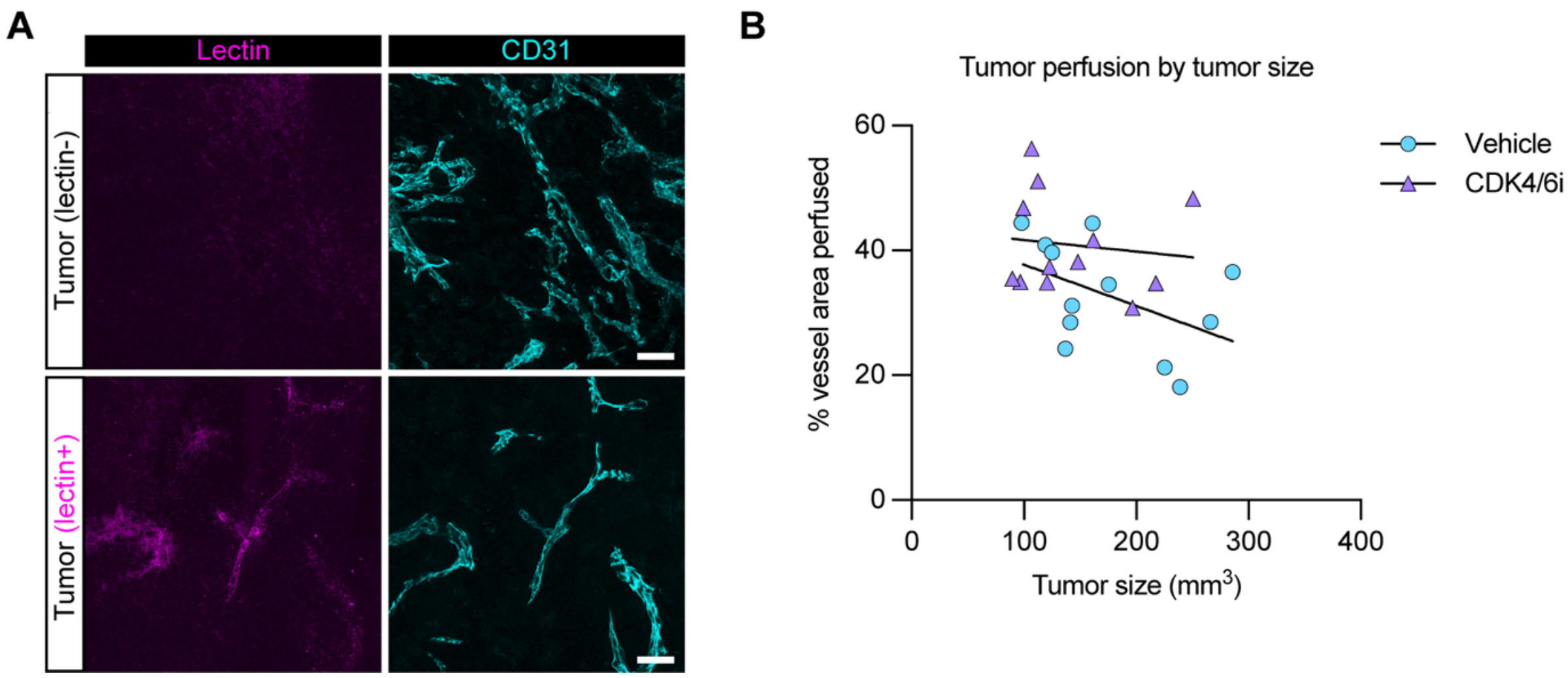

Supplemental Figure 8: Relationship between EO771 tumor volume and vessel perfusion. (**A**) Confocal images of lectin negative control (top) compared to lectin-perfused mouse (bottom) IF-stained for CD31 and used to set parameters for image acquisition. Scale bars = 50 µm. (**B**) Linear regression analysis of tumor volume (mm^3) vs. percent vessel area perfused. Vehicle R squared = 0.2165 and CDK4/6i treated R squared = 0.0148. Line slopes not significantly different (*p*=0.4492), line elevation is approaching significance (*p*=0.0682). Statistics calculated with Prism version 10.5.1 simple linear regression analysis of tumor volume (mm^3) vs. perfusion.

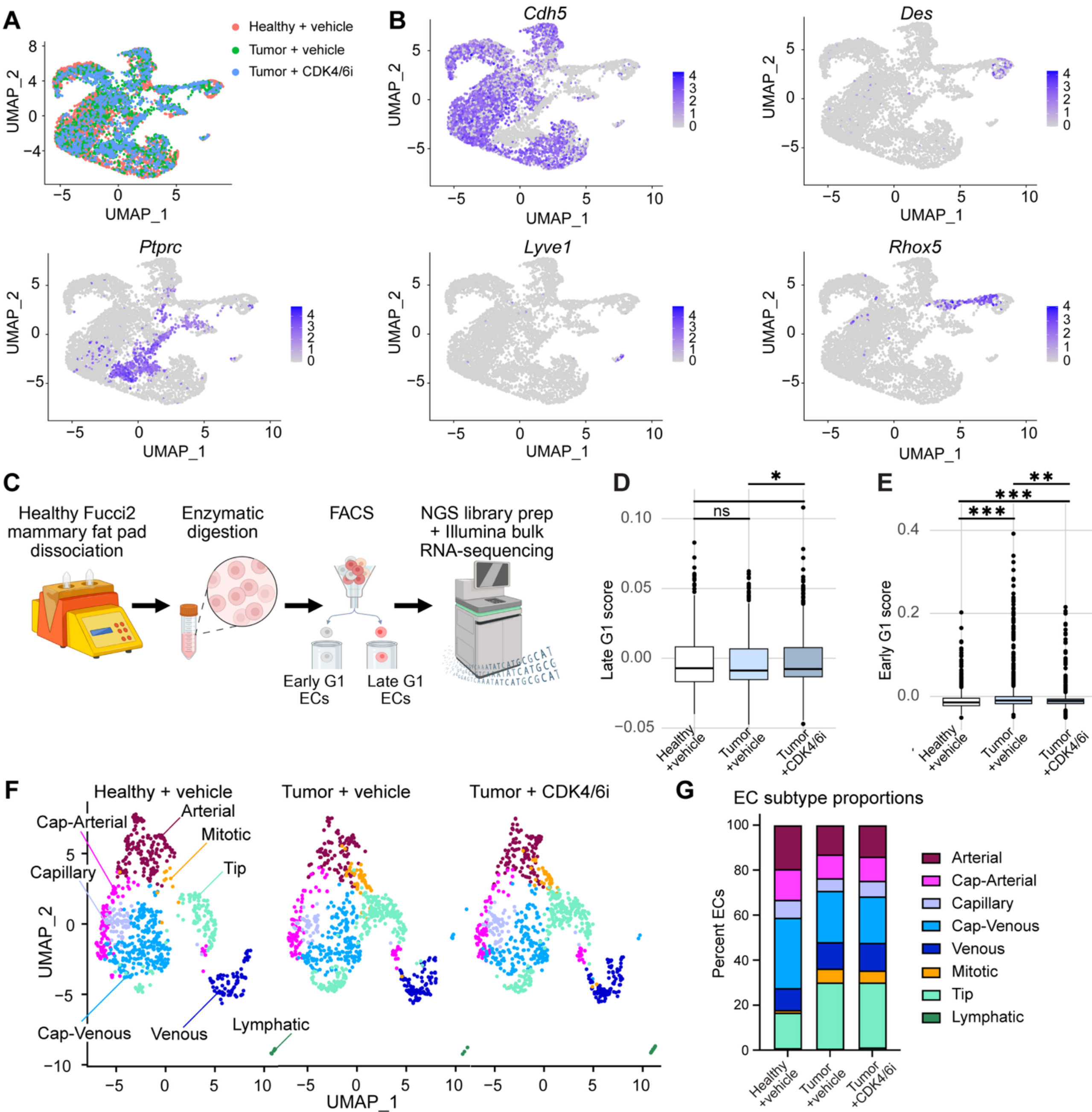

Supplemental Figure 9: **Tumor cell subtypes and tumor EC subtype proportions and cell cycle scores.** (**A**) UMAP colored by original identity of healthy, tumor, and CDK4/6i-treated tumor ECs and other cells. (**B**) UMAP feature plots with gene expression indicating different cell types: *Cdh5* (EC), *Des* (pericyte), *Ptprc* (immune), *Lyve1* (lymphatic ECs), and *Rhox5* (tumor cells). (**C**) Workflow for isolating NECs in early G1 and late G1 based on Fucci expression and subsequent RNA sequencing, created in BioRender. Cain, S. (2026) https://BioRender.com/3ltpb7b. Box plot of scRNAseq data module-scored for late G1 (**D**) and early G1 (**E**) gene signatures. Module scores were calculated using the top 90 genes enriched in late or early G1 identified by differential gene expression analysis of bulk RNA sequencing data of NECs in early vs. late G1. The black center line in the box is the median, with the box encompassing the 25th-75th percentiles. Whiskers extend to 1.5x the interquartile range. (**F**) EC subtypes represented with UMAP dimensionality reduction and split by tissue sample type and treatment. (**G**) Proportions of EC subtypes within NECs, TECs, and CDK4/6i-treated TECs. Statistical comparisons were performed in R (V4.4) with ggplot2 and ggpubr packages using the Kruskal-Wallis test followed by the Wilcoxon rank sum test to calculate *p*-values for pairwise comparisons with post hoc Holm correction (D-E); ***p<0.001, **p<0.01, *p<0.05, ns = nonsignificant.

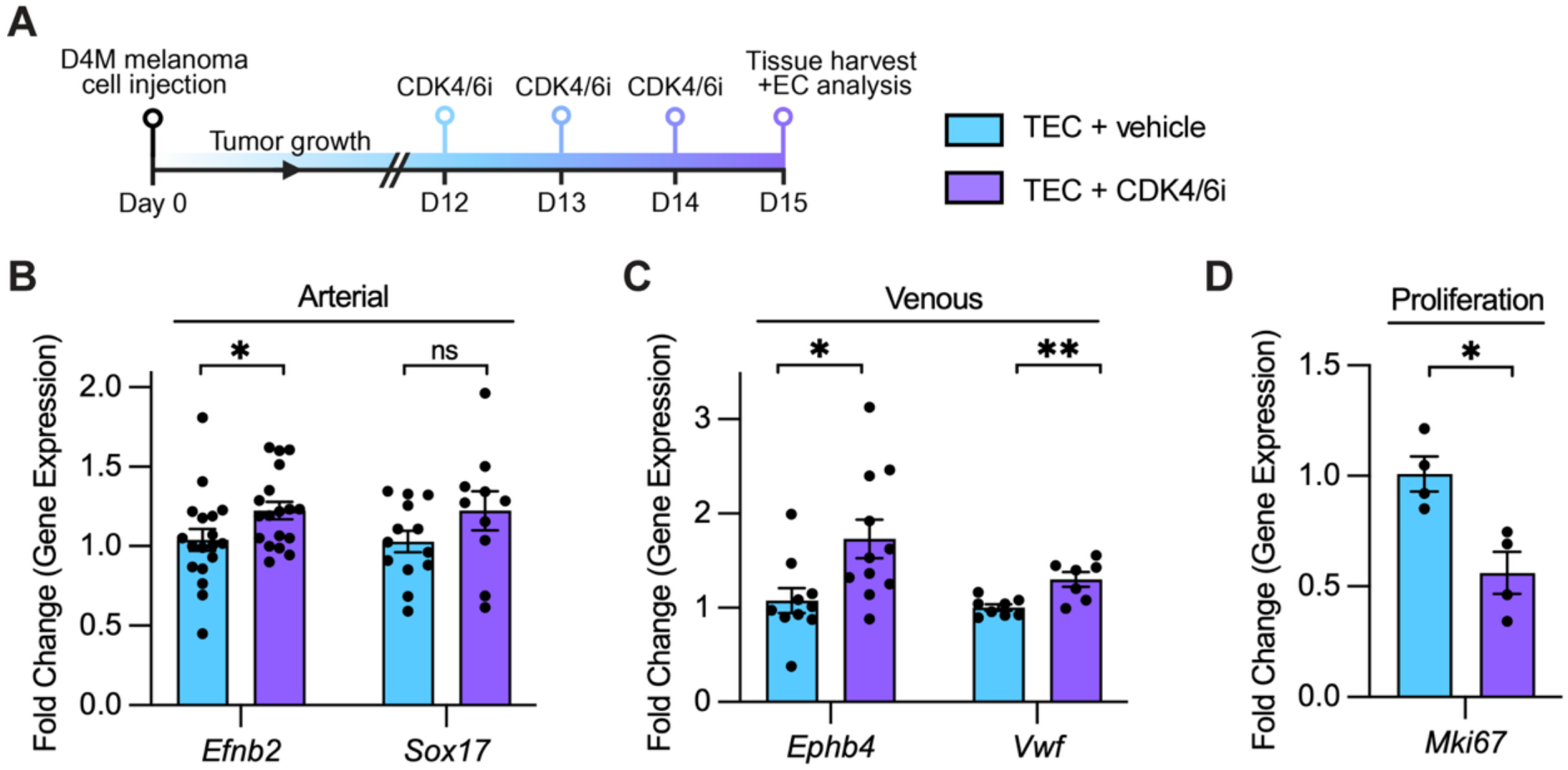

Supplementary Figure 10: Palbociclib increases arterial-venous gene expression in D4M melanoma. (**A**) Experimental timeline for mouse D4M melanoma model with CDK4/6 treatment, created in BioRender. Cain, S. (2026) https://BioRender.com/o6mt08u. qPCR analysis of arterial (**B**), venous (**C**), and proliferation gene (**D**) mRNA expression in ECs isolated from D4M tumors±CDK4/6i. Unpaired nonparametric Mann-Whitney statistical test. All data are expressed as mean±SEM; ***p*<0.01, *p<0.05, ns=nonsignificant.

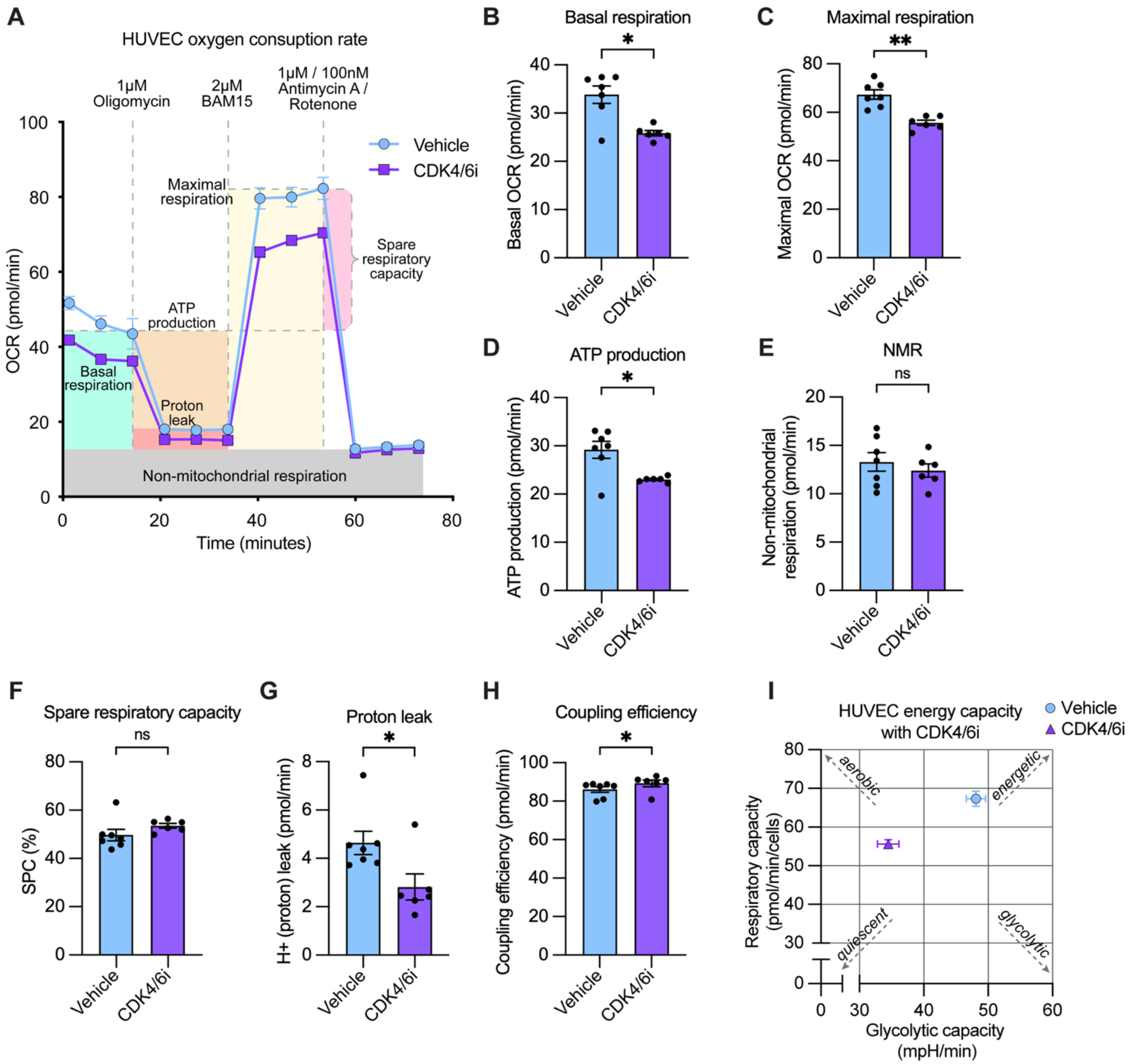

Supplementary Figure 11: Palbociclib suppresses mitochondrial respiration in HUVECs. (**A**) Representative traces of HUVEC oxygen consumption rate (OCR) $\pm$CDK4/6i pretreatment for 24 hours (n=6-7), during mitochondrial stress test (Seahorse XF24 and X96 Flux Analyzer) and labeled with injection points: oligomycin (1µM; ATP synthase inhibitor), BAM15 (2µM; mitochondrial uncoupler), and Antimycin A/rotenone (1µM/100nM; mitochondrial electron transport chain inhibitors). Basal respiration (green), ATP production (orange), proton leak (red), maximal respiration (yellow), spare respiratory capacity (pink). (**B**) Basal respiration measured as basal OCR minus non-mitochondrial OCR. (**C**) Maximal respiration is the maximum OCR following BAM15 treatment minus non-mitochondrial OCR. (**D**) ATP production is basal respiration minus OCR following oligomycin injection. (**E**) Non-mitochondrial respiration is OCR following Antimycin A and rotenone injection. (**F**) Spare respiratory capacity is the difference between maximal and basal respiration. (**G**) Proton leak is the difference between OCR following oligomycin and non-mitochondrial respiration. (**H**) Coupling efficiency is ATP production rate / basal respiration rate x100. (**I**) Energy capacity map displaying the shift in respiratory and glycolytic metabolic activity following CDK4/6i treatment. *P*-values were calculated with unpaired nonparametric Mann-Whitney statistical tests were using Prism version 10.5.1. All data are expressed as mean$\pm$SEM; ***p*<0.01, **p*<0.05, ns=nonsignificant.

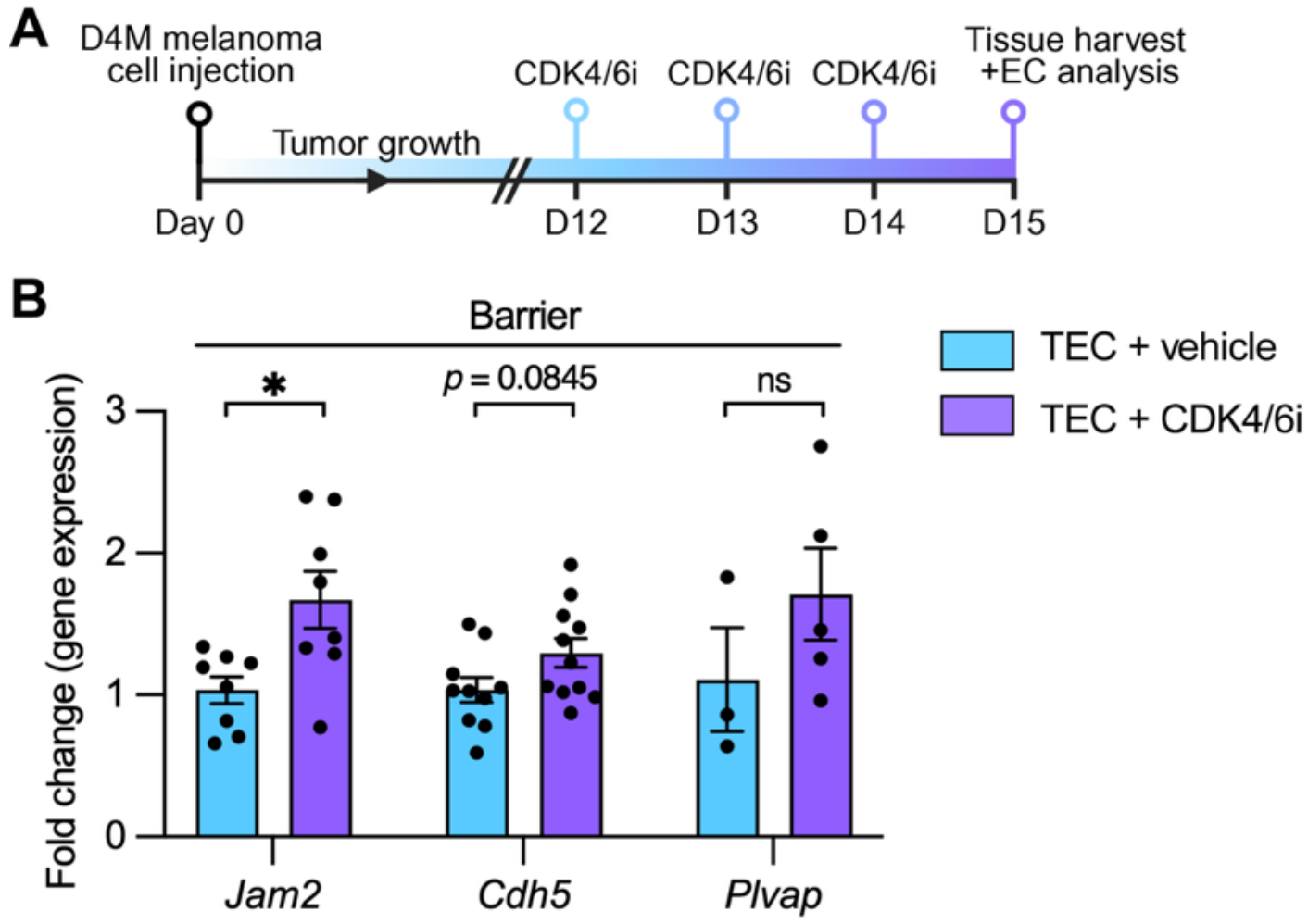

Supplemental Figure 12: Expression of barrier genes in D4M TEC following CDK4/6i treatment. (**A**) Experimental timeline for mouse D4M melanoma model with palbociclib (CDK4/6i) treatment, created in BioRender. Cain, S. (2026) https://BioRender.com/7qmlhtt (**B**) *Jam2*, *Cdh5*, and *Plvap* mRNA expression in TECs isolated from D4M tumors ±CDK4/6i (n=8). Unpaired nonparametric Mann-Whitney statistical test. All data are expressed as mean±SEM; *p<0.05, ns = not statistically significant.

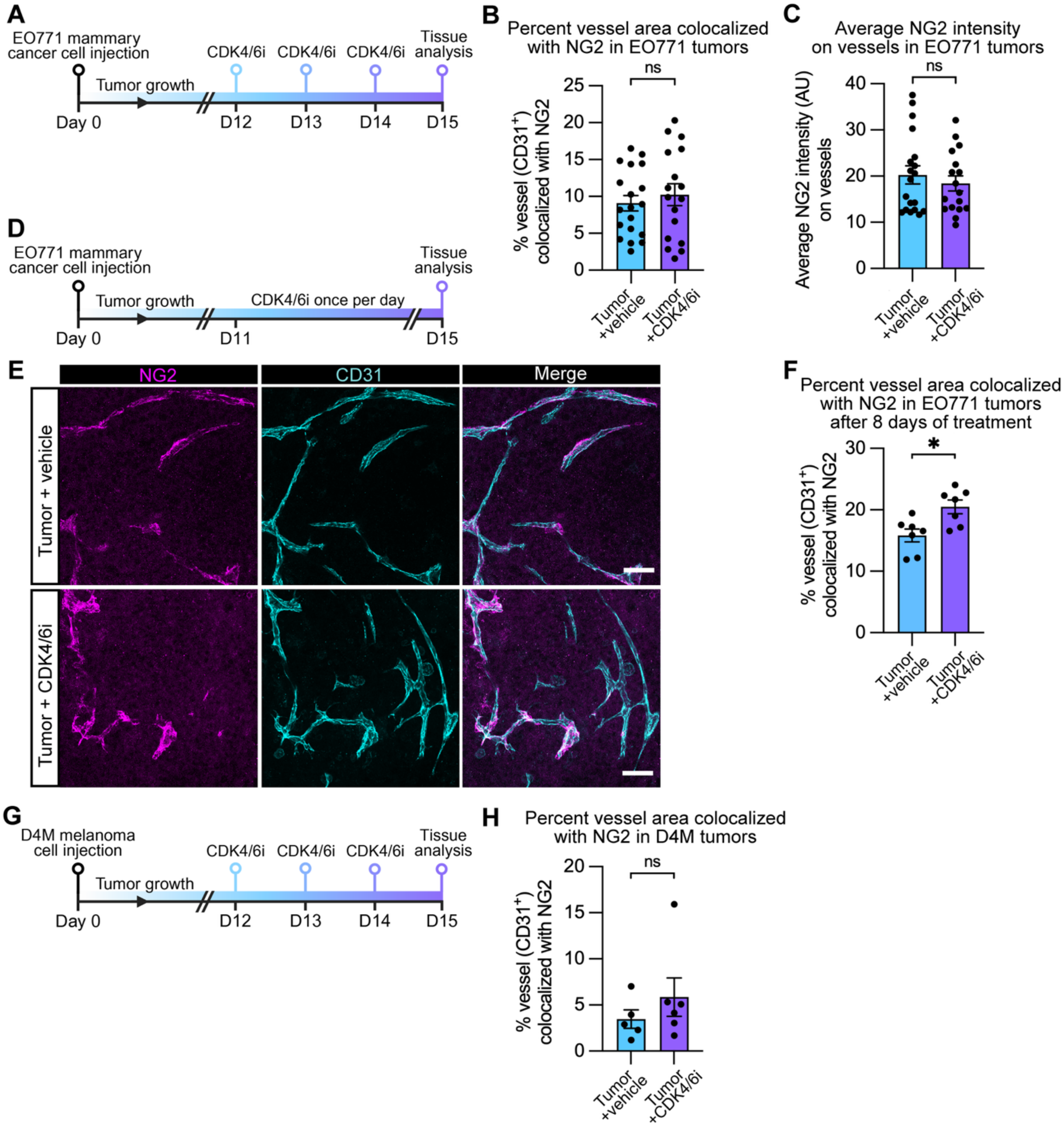

Supplemental Figure 13: **Vessel pericyte coverage in EO771 and D4M tumors following CDK4/6i.** (**A**) Experimental timeline for EO771 mouse mammary cancer model with CDK4/6 treatment at 150mg/kg/day for 3 days. (**B**) Quantification of average percent pericyte coverage per tumor, vessels ($CD31^+$) were analyzed for colocalization with contiguous NG2 signal. (**C**) Quantification of NG2 signal intensity in vessel regions of interest, also quantified using custom ImageJ macros. In 4 replicate experiments, 4-8 images per tumor were analyzed with custom ImageJ macros that identified vessel regions of interest for quantifications (n=17-19 mice total). (**D**) Experimental timeline for EO771 mouse mammary cancer with CDK4/6i treatment at 150mg/kg/day for 8 days. (**E**) Representative confocal images of pericytes (NG2) and vessels (CD31) in vehicle- (top) or CDK4/6i- (bottom) treated tumors. (**F**) Quantification of average percent vessels (CD31) colocalized with contiguous NG2 per tumor using the custom ImageJ macro described in (B). n=7 mice, 3-6 images analyzed per tumor. (**G**) Experimental timeline for D4M melanoma tumor model treated daily with CDK4/6i (150mg/kg) for 3 days. (**H**) Quantification of average percent vessel area ($CD31^+$) colocalized with contiguous pericyte coverage (NG2 signal) per tumor (n=5-6). *P*-values were calculated with unpaired nonparametric Mann-Whitney statistical tests were using Prism version 10.5.1. All data are expressed as mean±SEM; **p*<0.05, ns=nonsignificant. (A), (D), and (G) created in BioRender; Cain, S. (2026) https://BioRender.com/q6dd9vi.

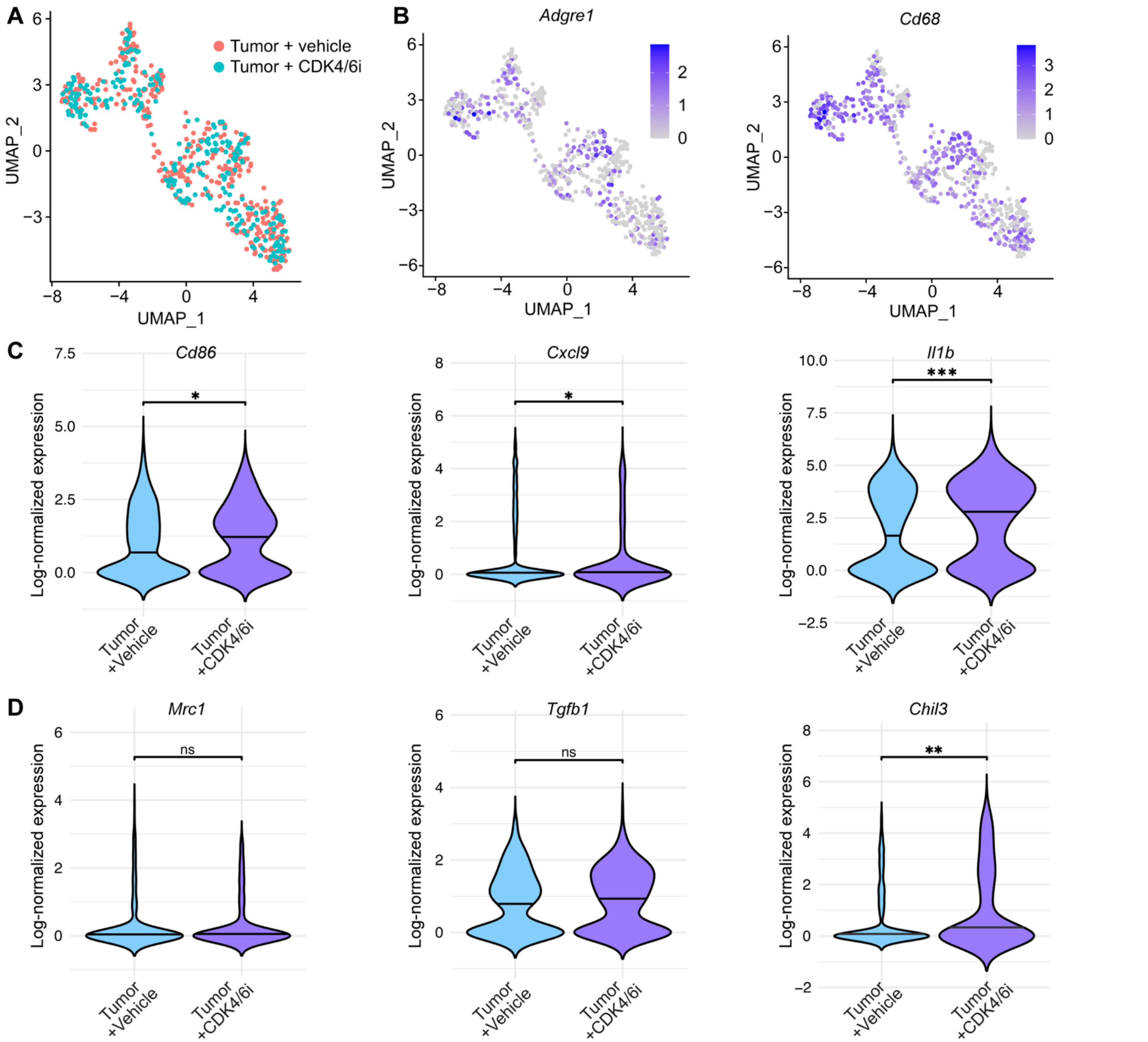

Supplemental Figure 14: Macrophage polarization-associated gene analysis. (**A**) UMAP colored by original identity of tumor and CDK4/6i-treated tumor macrophages. (**B**) UMAP feature plots with gene expression indicating macrophage identity: *Cd68*, *Adgre1*. Violin plot of log-normalized mRNA expression associated with M1 (**C**) and M2 (**D**) polarization in macrophages from vehicle- vs. CDK4/6i-treated tumors. Medians are represented by black horizontal line on the plots. Statistical comparisons were performed in R (V4.4) with ggplot2 and ggpubr packages using the Kruskal-Wallis test followed by the Wilcoxon rank sum test to calculate *p*-values for pairwise comparisons with post hoc Holm correction (C-D); ****p*<0.001, ***p*<0.01, **p*<0.05, ns=nonsignificant.

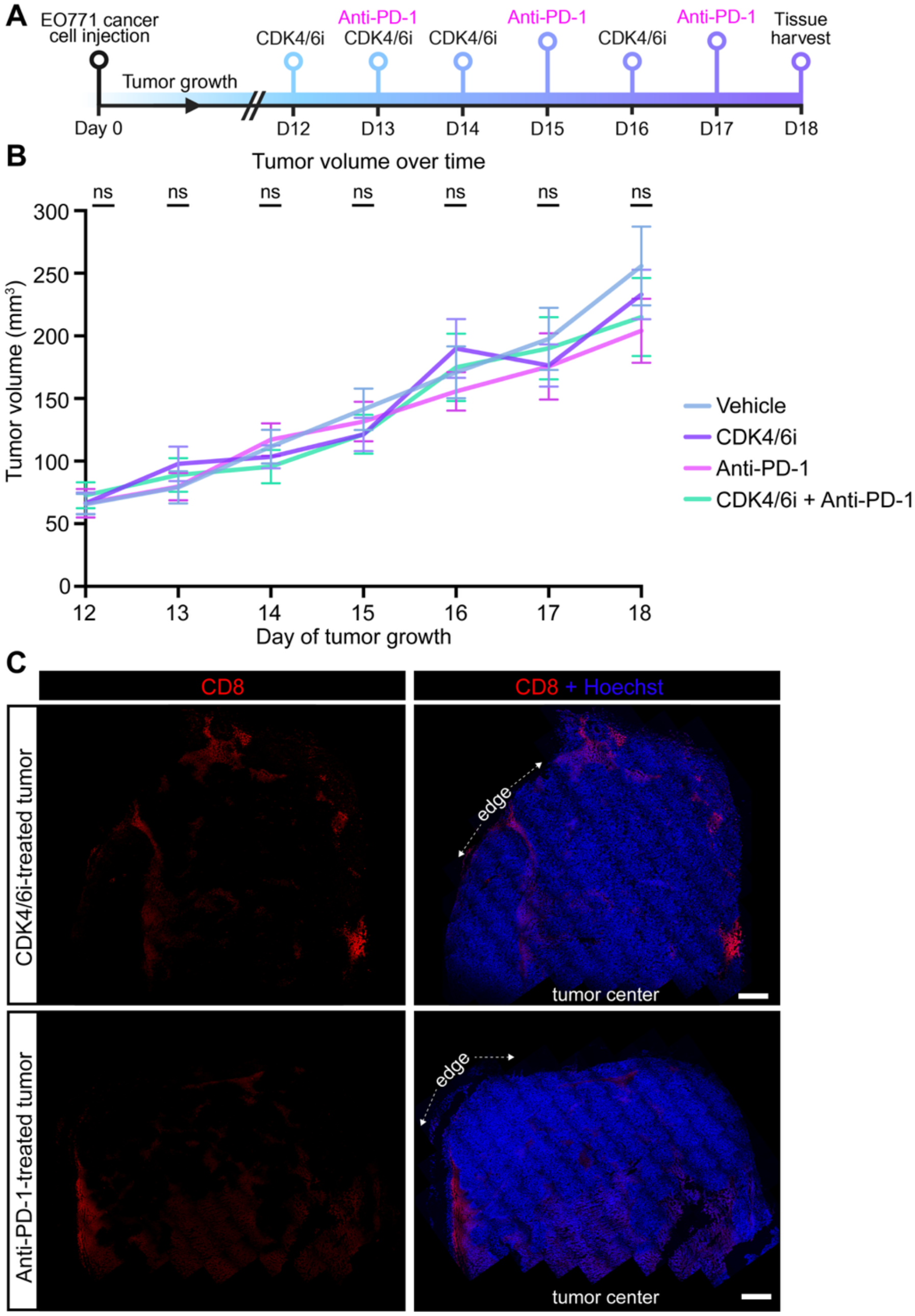

Supplemental Figure 15: **Immune signaling in EO771 tumors.** (**A**) Experimental design for tumor model with groups receiving vehicle control only, CDK4/6i monotherapy (150mg/kg/day, oral gavage), anti-PD-L1 monotherapy (15mg/kg, IP injection) or combination. CDK4/6i vehicle is 10% DMSO in PBS; anti-PD-1 vehicle is IgG2a (15mg/kg) in PBS, created in BioRender. Cain, S. (2026) https://BioRender.com/1ho1tvx. (**B**) Tumor size (mm^3) over time during CDK4/6i and anti-PD-1 treatments. Repeated measures two-way ANOVA performed with Šídák's multiple comparisons test (n=10-11). All data are expressed as mean±SEM; ns=nonsignificant. (**C**) Representative confocal images of tumor cross section immunofluorescent-stained for CD8 (red) and nuclei labeled with Hoechst (blue) following CDK4/6i (top) or anti-PD-1 (bottom) monotherapy treatment. Images taken from edge of tumor to the center, labeled in white text. Scale bar = 500µm.

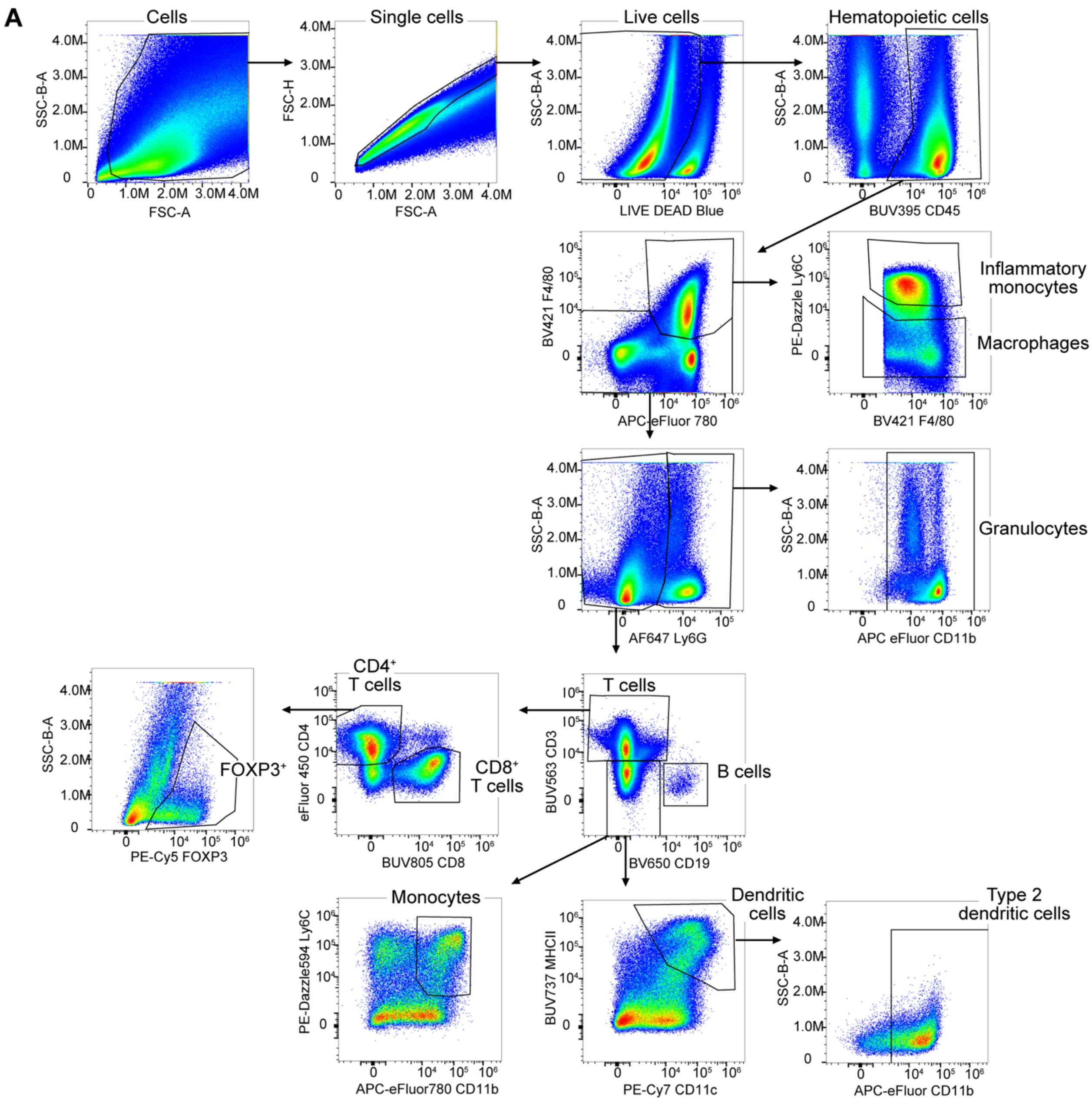

Supplemental Figure 16: **Flow cytometry gating strategy for immune cell analysis.** (**A**) Cells were first gated for single cells, live cells (Blue-) and hematopoietic cells (CD45$^+$). The following immune populations were identified: inflammatory monocytes (F4/80$^+$CD11b$^+$Ly6C^{high}), macrophages (F4/80$^+$CD11b$^+$Ly6C$^-$), granulocytes (Ly6G$^+$CD11b$^+$), dendritic cells (F4/80$^-$CD11c$^+$MHCII$^+$), monocytes (F4/80$^-$CD11b$^+$Ly6C$^+$), type II dendritic cells (F4/80$^-$CD11c$^+$MHCII$^+$CD11b$^+$), B cells (F4/80$^-$Ly6G$^-$CD3$^+$CD19$^+$), T cells (F4/80$^-$Ly6G$^-$CD3$^+$CD19$^-$) and within T cells, regulatory T cells (CD4$^+$FoxP3$^+$) and cytotoxic T cells (CD8$^+$).

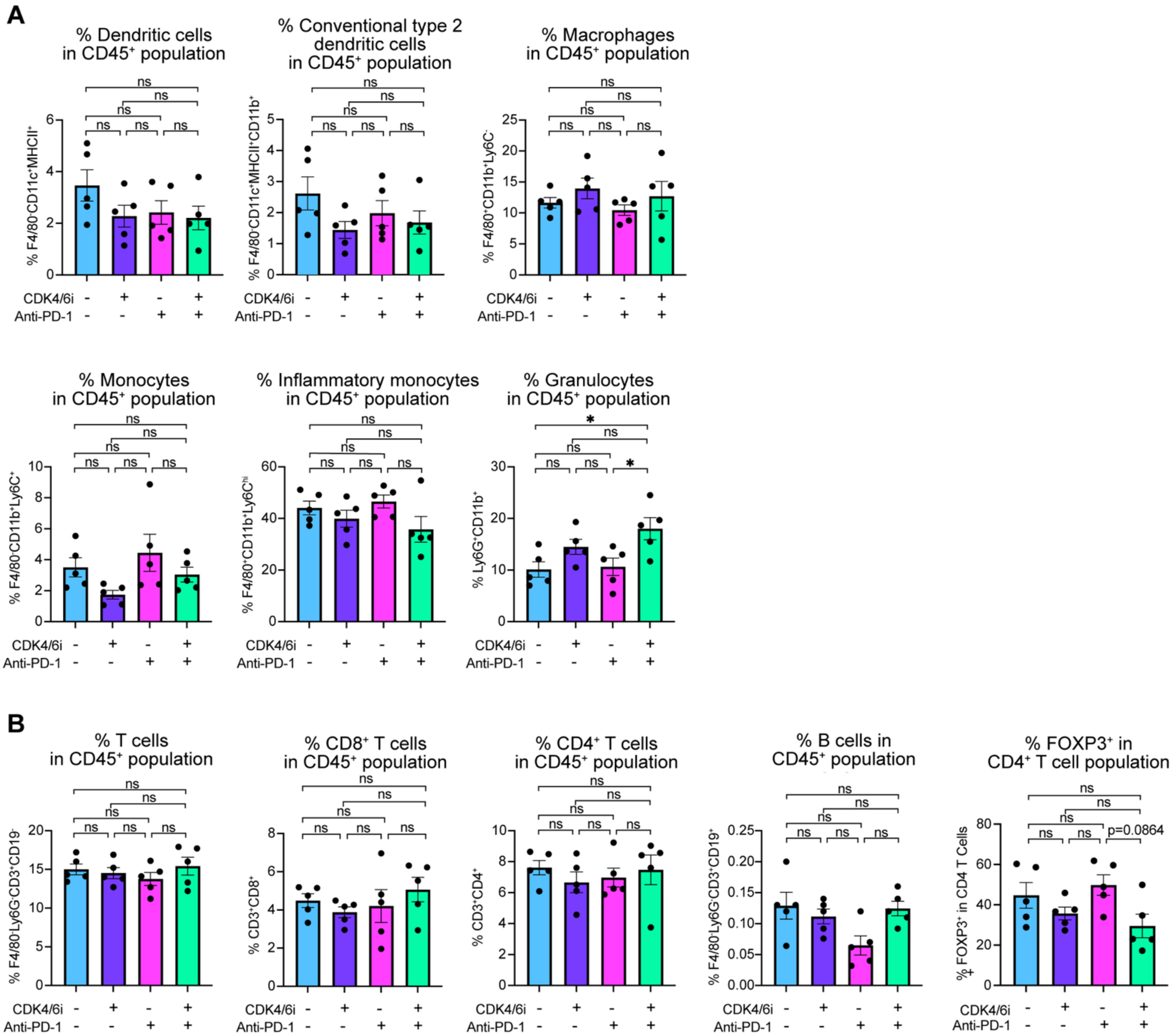

Supplemental Figure 17: **Flow cytometry immune cell quantifications. A.** Percent myeloid cell subtypes in CD45$^+$ population (n=5). **B.** Percent lymphoid subtypes in CD45$^+$ population (n=5). Statistical tests two-way ANOVA and Šídák's multiple comparisons test, all data are expressed as mean$\pm$SEM; **p*<0.05, ns=nonsignificant.

Supplemental Table 1: **NEC gene expression in early G1 vs late G1**. See supplemental spreadsheet. The top 90 most enriched genes in early G1 and late G1 identified through bulk RNAseq of Fucci-expressing NECs that were also found in the scRNAseq Seurat object were used to assign cell cycle module scores.

Name	Sequence (5'-3')
mVenus forward	GAAGCAGCACGACTTCTTCA
mVenus reverse	GACGTTGTGGCTGTTGTAGTTG
Internal positive control forward	CTAGGCCACAGAATTGAAAGATCT
Internal positive control reverse	GTAGGTGGAAATTCTAGCATCATCC

Supplemental Table 2: Primers for global Fucci2-expressing mice. Forward and reverse primers for mVenus to identify Fucci2^{+} mice with internal positive control forward and reverse primers.

Marker	Clone	Host	Supplier	Catalogue#	Dilution
ERG	EPR3864	Rabbit	Abcam	ab92513	1:100
GFP	polyclonal	Goat	Origene	R1091P	1:100
mCherry	polyclonal	Chicken	Origene	TA150127	1:100
EMCN	V.7C7	Rat	ThermoScientific	14-5851-82	1:100
CD8a	53-6.7	Rat	ThermoScientific	14-0081-82	1:100
CD31	2H8	Armenian hamster	ThermoScientific	MA3105	1:100
CD31	MEC13.3	Rat	BD Biosciences	550274	1:100
NG2	546930	Rat	R&D	MAB6689	1:100
BST2	120G8.04	Rat	Novus Biologicals	DDX0390P-100	1:100

Supplemental Table 3: Primary antibodies used for immunofluorescence staining in tissues. Antibody target antigen, specific antibody clone, catalogue information, and dilutions listed were used for tissue analysis.

Target	Fluorophore	Supplier	Catalogue#	Dilution
Rat	AF647	Abcam	ab150155	1:200
Armenian hamster	AF488	JAX	127-545-160	1:200
Rabbit	AF647	JAX	711-605-152	1:200
Goat	AF488	Abcam	ab150133	1:250
Chicken	AF594	JAX	703-585-155	1:250
Rat	AF568	Abcam	ab175475	1:200
Armenian hamster	AF647	ThermoScientific	A78967	1:200

Supplemental Table 4: Secondary antibodies used for immunofluorescence staining in tissues. Antibody target species, fluorophore, catalogue information, and dilutions listed were used for tissue analysis.

Marker	Fluorophores	Clone	Supplier	Catalogue #	Dilution
CD31	PE	MEC 13.3	BD Biosciences	553373	1:100
CD45	PerCP	30 F11	BD Biosciences	557235	1:100
CD31	BV421	MEC 13.3	BD Biosciences	562939	1:100
CD45	PE-Cy7	30 F11	BD Biosciences	552848	1:100
CD45	BUV395	30-F11	BD Biosciences	565967	1:400
CD11b	APC-eF780	M1/70	Thermo Fisher	47-0112-82	1:1000
F4/80	BV421	BM8	BioLegend	123131	1:100
Ly6C	PE Dazzle 594	HK1.4	BioLegend	128043	1:2000
Ly6G	AF647	1A8	BioLegend	127609	1:500
CD3	BUV563	145-2C11	BD Biosciences	749277	1:100
CD4	eF450	RM4-5	Thermo Fisher	48-0042-82	1:2000
CD8	BUV805	53-6.7	Thermo Fisher	368-0081-82	1:250
CD11c	PE/Cy7	N418	Invitrogen™	25-0114-82	1:1000
MHCII	BUV737	M5/114.15.2	BD Biosciences	748845	1:400
CD19	BV650	1D3	BD Biosciences	563235	1:500
FOXP3	PE-Cy5	236A/E7	ThermoFisher	15-4777-42	1:50

Supplemental Table 5: Fluorescent antibodies used for immune flow cytometric studies. Antibody target, fluorophore are listed along with specific antibody clone, catalogue information, and dilutions used for immune cell analysis.
